# Toll of Chronic Metabolic Acidosis at Molecular, Cellular, and Systemic Levels: A Conceptual Framework to Revisit Type 2 Diabetes (T2D) Pathophysiology

**DOI:** 10.3390/biomedicines14040901

**Published:** 2026-04-15

**Authors:** Mai S. Sater, Hayder A. Giha

**Affiliations:** 1 Department of Medical Biochemistry, College of Medicine and Health Sciences (CMHS), Arabian Gulf University (AGU), Manama 26671, Bahrain; 2 Centre for Cardiovascular Science, Queens Medical Research Institute (QMRI), College of Medicine, University of Edinburgh, 47 Little France Crescent, Edinburgh EH16 4TJ, UK

**Keywords:** type 2 diabetes, chronic metabolic acidosis (CMA), T2D pathophysiology, pH homeostasis, insulin resistance, acid-neutralization therapy

## Abstract

**Background/Objectives**: Chronic metabolic acidosis (CMA) is a mild, persistent acid–base imbalance characterized by low serum bicarbonate and urinary pH and is common in chronic illness, aging, and metabolic disorders such as type 2 diabetes (T2D). This review highlights the critical, yet often overlooked, role of CMA in T2D (CMAD) and its contribution to disease pathophysiology. **Methods**: We conducted a comprehensive review of the systemic impacts of CMA, from molecular mechanisms to organ-specific dysfunction. The analysis covers physiological pH dynamics in intracellular (IC) and extracellular (EC) fluids and explores their effects on cellular processes, including the cell cycle and apoptosis. **Results**: At the molecular level, acidosis significantly alters enzyme kinetics, macromolecule metabolism, and ion conductance. Cell-level analysis shows that pH shifts impact proliferation and programmed cell death. Systemically, the manifestations of CMA align closely with T2D features in vital organs, including the pancreas, liver, skeletal muscle, adipose tissue, and the renal, nervous, and immune systems. Our findings indicate that the pathophysiological landscape of T2D largely mirrors the biological effects of chronic acidosis. **Conclusions**: The alignment between the effects of CMA and the clinical features of T2D suggests that T2D pathophysiology is worth revisiting through the lens of CMAD. This perspective is further supported by therapeutic interventions showing preliminary efficacy signals in limited studies of acid-neutralization in managing T2D symptoms and progression.

## 1. Introduction

Type 2 diabetes (T2D) is a chronic metabolic condition that has reached pandemic proportions, with global prevalence in adults estimated to reach 700 million by 2045. It accounts for more than 90% of all diabetes cases worldwide [1]. It is characterized by insulin resistance (IR) and relative insulin deficiency, leading to chronic hyperglycemia, which, if unmanaged, causes severe, long-term damage to major organs [1]. T2D is a highly heterogeneous metabolic disorder characterized by diverse genetic and environmental etiologies and complex pathophysiology [2]. Acidosis represents a major metabolic abnormality in T2D, recently termed chronic metabolic acidosis of diabetes (CMAD) [3]. Whether T2D causes CMAD or vice versa remains unclear, though these possibilities are not mutually exclusive; a vicious cycle likely perpetuates both conditions chronically. Several studies have documented the relationship between metabolic acidosis and IR [4,5,6], demonstrating that individuals with high-anion-gap metabolic acidosis and low serum HCO_3_ exhibit reduced insulin sensitivity [5]. Furthermore, IC acidosis substantially disrupts mediators downstream of the PI3K (phosphatidylinositol-3 kinase)/Akt (serine/threonine kinase) signaling pathway, which normally stimulates translocation of glucose transporter 4 (GLUT4) to the cell membrane and promotes glucose uptake in target tissues, thereby reducing glucose uptake efficiency and causing IR [6]. Conversely, IR, a cardinal feature of T2D, likely contributes to chronic metabolic acidosis by diverting glucose metabolism toward anaerobic pathways and lactic acid production at the expense of aerobic energy generation [7,8].

The human body maintains strict pH control across cellular compartments through compensatory mechanisms that resist pH changes in both ECF and ICF. When compensatory capacity is exceeded, diverse acid–-base disorders emerge. The lungs and kidneys serve as the final routes for proton extrusion from the body [9,10]. In the setting of increased body acidity in T2D, while most tissue and organ fluids experience acidification, at least three sites exhibit increased alkalinity: gastric contents, pancreatic β-cells, and the skin surface [3]. However, this alkalinity also contributes to the pathophysiology, symptoms, and complications of T2D, as discussed below. Both the acidity and alkalinity of the surrounding medium affect an enzyme’s catalytic activity, primarily by altering its 3-dimensional structure and active-site conformation. As previously noted, CMAD in T2D may be driven by mitochondrial dysfunction, inflammation, gut microbiota dysbiosis, and diabetic lung disease [3].

## 2. Normal Body pH

A wide pH variation exists between different body compartments, such as the ICF and ECF (Table 1). Controlling the pH of ECF and ICF is crucial for optimizing biochemical reactions and maintaining tissue homeostasis. For simplicity, the ECF is categorized into blood, interstitial fluid (ISF), and specific body fluids, such as pleural, pericardial, peritoneal, synovial, and cerebrospinal fluids, as well as secreted and excreted fluids such as saliva, sweat, tears, seminal fluid, and urine. The lowest pH is found in gastric secretions (pH 1–3.5), while the highest occurs in pancreatic secretions (pH 8.0–8.3). Excretions like urine, seminal fluid, and feces exhibit a broad normal pH range (4.6–8.4) (Table 1). Modified pH reference ranges for various body fluids in T2D patients may be necessary, as their bodies adapt to slightly increased acidity with the resetting of compensatory systems. This adjustment is especially important for drug therapies, which are highly sensitive to pH.

**Physiological pH of the ICF (cytoplasm) and organelles:** The IC pH (pHi) of living cells is predominantly maintained at alkaline levels, with an overall range of approximately 7.03–7.46, which is wider than that of arterial (pH 7.35–7.45) and venous (pH 7.32–7.43) blood [11]. The pHi is maintained primarily by protein and phosphoric acid (H_3_PO_4_) buffers, while histidyl dipeptides serve as major buffers in skeletal muscle. Several membrane transporters responsible for proton removal from the cytosol play important roles in maintaining alkaline pHi [8].

pH variations exist not only between cell types in terms of cytosolic pH (pHi) (Table 2) [11], but also between organelles within the same cell (pH 4.5 to 8.0) and even within different regions of the same organelle, such as mitochondria and the Golgi apparatus. Nevertheless, pHi in each cell type is strictly maintained within a narrow range of <0.1 pH units [12]. The pH within each organelle is tailored to its specific function. For example, lysosomes maintain a relatively low pH of 4.5 [31], as their degradative function requires high acidity [32]. In contrast, the mt matrix has a pH of approximately 8.0, which is roughly 0.9 pH units higher than the intermembrane space of the same mitochondrion [31,33]. This pH gradient is necessary to create the electrochemical gradient across the membrane required for oxidative phosphorylation. Furthermore, pH decreases along the cis-trans axis of the Golgi apparatus from 6.7 (cis-Golgi) to 6.0 (trans-Golgi network) [34].

Parenchymal cells of two organs, myocytes (muscle) and hepatocytes (liver), play central roles in IR and serve as useful examples for discussing the role of ion transporters in maintaining pHi within the normal range. In active muscle, over 80% of myocyte IC protons are transported to the ECF via monocarboxylate transporters (MCTs), while the remaining 20% are transported via the Na^+^/H^+^ exchanger (NHE), HCO_3_^−^-coupled transporters, and H^+^-coupled transporters [45]. Thus, regulation of proton transporters is closely associated with pH maintenance capacity [46]. In the liver, in addition to common IC sources of acid, protons are generated from organic acids such as ketone bodies (KBs); consequently, pHi can be easily disturbed [47] unless maintained by buffering systems and proton efflux, predominantly through MCTs that extrude protons across the plasma membrane. However, the compensatory mechanisms for acid–base balance, including buffers and ion transporters for each tissue type, represent an extensive topic addressed elsewhere [3].

**Physiological pH of interstitial fluids (ISF)—pHn:** H^+^ produced during cellular metabolism is transported via two pathways: (1) Direct transport of H^+^ from the IC to the IS space by H^+^ transporters such as NHE and H^+^-ATPase, and (2) indirect transport whereby H^+^ combines with HCO_3_^−^ to form H_2_CO_3_, which dissociates into CO_2_ and H_2_O. CO_2_ exits the cell into the ISF, where it reconstitutes H_2_CO_3_ and subsequently releases H^+^ into the IS space [48]. The HCO_3_^−^ consumed in the IC space is replenished by IS HCO_3_^−^ transported from the IS space via HCO_3_^−^ transporters such as NDCBE (sodium-driven chloride/bicarbonate exchanger) and NBC (Na^+^/HCO_3_^−^ cotransporter). The ISF plays a critical role in transmitting EC signals through ligands, including hormones and neurotransmitters, that regulate IC functions, including metabolism. Since most ligands are proteinaceous in nature, alterations in the interstitial pH (pHn) affect signal transduction efficiency, directly impacting cell function and homeostasis.

### 2.1. The Consequences of Chronic Metabolic Acidosis

Changes in pH have a marked effect on all reactions catalyzed by protein enzymes, since each enzyme has a specific pH optimum. The pH is the major determinant of ionization of the amino acid components of the proteins and thus their conformation and activity. However, the IC environment is largely protected by strong buffering systems that transfer the H^+^ or HCO_3_^−^ surplus to the EC compartment. In T2D, it is likely that chronic acidosis skips correction with consequent local and systemic metabolic and pathological effects. In the following sections, we briefly present the systemic consequences of acidosis in general and show their similarity to the features of T2D.

### 2.2. Systemic and Metabolic Effects of Acidosis

The systemic effects of acidosis that will be discussed here are those that occur at the a. cellular/tissue level (cell cycle, apoptosis) and b. molecular level (metabolic/enzymatic, ion transport).

**A. Effects of acidosis at the cellular/tissue level:**

**Figure 1:**
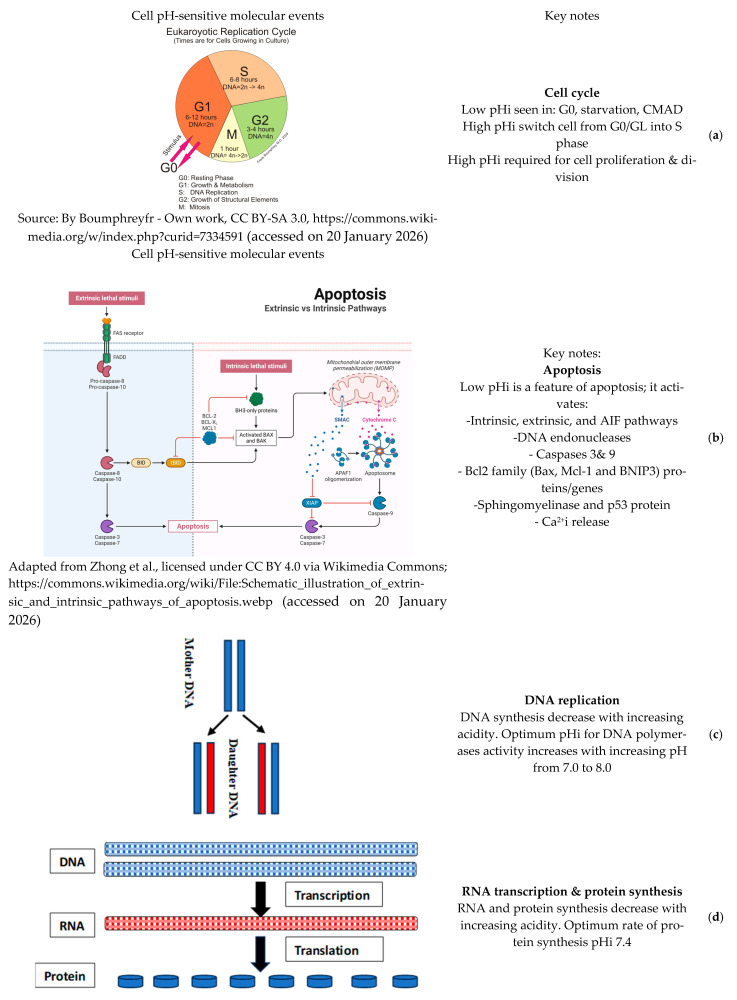

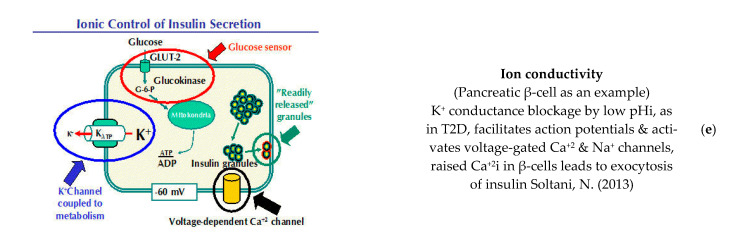
Illustrations of cellular mechanisms and molecular pathways influenced by pH: (**a**) cell cycle, (**b**) apoptosis pathways, (**c**) DNA replication, (**d**) RNA transcription/protein synthesis, and (**e**) ion conductivity in pancreatic β-cells, along with the major effects of pHi levels on these essential processes [58].

Cytoplasmic pH has been shown to play a crucial role in multiple cellular functions, including cell growth and proliferation, and programmed cell death (apoptosis) [49]. Although these effects are mostly due to acidosis, there is evidence that IC alkalization can also induce apoptosis [50].

i. Effects pH on the cell cycle: Oscillations in pHi have been linked to the control of the cell cycle and cell division in several cell types. Low pHi is common in resting cells, which coincides with a low cellular metabolic profile, while the rapid increase in pHi brings cells from G0/GL into the S (synthesis) phase of the cell cycle (Figure 1a). During the cell cycle, the pH increases to 7.4–7.5 within one hour before mitosis [11]. Starvation of the cells, on the contrary, lowers the pH, interrupting the cell cycle by inhibiting mitosis. An increase in pHi can also explain the reactivation of quiescent cells to enter the cell cycle, i.e., the G0-to-G1 transition and proliferation. Therefore, a strictly controlled pHi is considered a second messenger for cellular growth control [11]. In T2D, it is well known that cell cycle and growth are disrupted across tissues, attributed to hyperglycemia and oxidative stress [51]. However, both are in crosstalk with CMAD. In T2D, in skeletal muscle, pHi decreases slightly, leading to protein aggregation and activation of autophagy [40], with concomitant cellular senescence and muscle atrophy [52]. In contrast, pHi increases in pancreatic β-cells, leading to loss of function and, later, impaired proliferation and dedifferentiation, resulting in pancreatic failure [53]. Nevertheless, the exact effect of CMAD on the cell cycle and proliferation is not reported in vivo or in vitro; however, the use of acid-neutralizing diets/therapy was shown to improve the growth of different cell types, including muscle and B-cell [54], which supports the influence of CMAD in the cell cycle suppression.

ii. Effects of pH on apoptosis: Apoptosis, also known as programmed cell death, is a physiological process necessary for tissue homeostasis and development. However, in pathological conditions, it leads to premature cell death. There are two main apoptotic pathways: the intrinsic (mt-dependent) and the extrinsic (death receptor) pathways. Both pathways activate caspases (a family of cysteine-aspartate-specific proteases) as a common endpoint (Figure 1b). In the mt pathway, the Bcl-2 family (including Bax, Bid, Bcl-xL, etc.) controls the release of cytochrome c (Cyt c) and other factors. Cyt c combines with Apaf-1 and procaspase-9 to form an “apoptosome,” which activates effector caspases such as caspase-3. The extrinsic pathway is triggered by EC factors such as infections/viruses, immune signals, hormones, and chemicals, acting through specific receptors collectively called death receptors. In addition to these two major pathways, there are caspase-independent apoptotic routes involving apoptosis-inducing factor (AIF) as a key regulator [55]. It should be noted that pH significantly influences all apoptosis pathways.

Cytosolic acidification is a common feature of apoptosis; however, its exact role remains unknown. It has been reported that DNase II (nuclear deoxyribonuclease II), which mediates the digestion of internucleosomal DNA characteristic of apoptosis, is activated in intact cells when pHi falls below pH 7.0 (acidification), independently of calcium [56]. Thus, the acidic role may be in apoptosis-related DNA degradation. Other low-pH-dependent endonucleases have been identified, including cytoplasmic DNase II precursor proteins, such as LEI (leucocyte elastase inhibitor), which is translocated into the nucleus upon activation [57].

Evidence supports the role of cytosolic acidification on caspases, specifically caspase 9 [59] and caspase 3 [60]. The optimal pH for caspase-9 activation via Cyt-c is 6.3–6.8 in vitro, and the assembly of the caspase activation complex, the apoptosome, may occur faster at acidic pH. The procaspase 3 silencing is attributed to a tripeptide Asp-Asp-Asp that is removed upon acidification, followed by maturation of procaspase 3, and hastening its proteolytic activation by caspase 9 [61]. Although alkaline pH inhibits caspase 3 activation, once activated, the protease’s activity remains unaffected by elevated pH [62]. It is also possible that pHi indirectly modulates caspase activity by regulating endogenous caspase-inhibitory proteins, such as inhibitors of apoptosis (IAPs), which suppress caspase 9 and 3 activity. Moreover, changes in pH affect the functions of Bcl-2 family proteins, through which acidosis favors apoptosis [50]. Besides affecting protein structure and function, acidification might also control the expression of the Bcl-2 gene family [63]. Bax proteins form pores in the mt membranes, thus allowing the release of proapoptotic molecules such as Cyt c, AIF, and smac-Diablo. It has been reported that the channel-forming activity of Bax is eight times greater at pH 4.0 than at pH 7.5 (physiological pH). Bcl-2 can completely block this pore-forming activity [64]. Regarding BNIP3 (BCL2/adenovirus E1B 19 kDa protein-interacting protein 3), it was shown that acidosis is necessary for this protein to induce apoptosis in hypoxic cardiac cells [65].

Other factors in the apoptotic cascade that are targets of cytosolic acidification include mt acid sphingomyelinase and p53 protein. The sphingomyelinase activation and associated ceramide production, following caspase 8 activation during Fas-induced apoptosis [66], are strongly enhanced by IC acidification. Additionally, p53 expression, as part of the cascade, may be sensitive to pHi changes. Other apoptotic signaling pathways that may be affected by cytosolic acidification include the p38 mitogen-activated protein kinase (MAPK) pathway [67]. Finally, the interactions between pHi and IC calcium (Ca^2+^i) homeostasis indicate the influence of cytosolic acidification on apoptosis, since it is well-documented that IC acidification increases Ca^2+^i [68].

On the other hand, apoptosis is one of the major features of T2D, and manifests in most vital tissues, contributing to the pathophysiology of the disease and its complications. Multiple studies have highlighted the effects of diabetic apoptosis in specific tissues, including pancreatic β-cell failure [69], muscle sarcopenia [70], neuropathy [71], liver and adipose tissue disorders [72], nephropathy [73], and micro/macro-vascularopathy [74]. In these studies, apoptosis in T2D was attributed to various factors, including mt dysfunction, IR, oxidative stress, ER stress, glucotoxicity, lipotoxicity, inflammation, and cytokines. Nevertheless, all of these factors are associated with increased acidity in T2D. It has been shown that apoptosis-related acidification is commonly due to mt dysfunction, a major feature of T2D [50]. Acidification might be responsible for mt dysfunction via mt membrane depolarization and the release of Cyt c [75]. Thus, it is tempting to speculate that CMAD is a causal factor of apoptosis in T2D.

**Figure 2:**
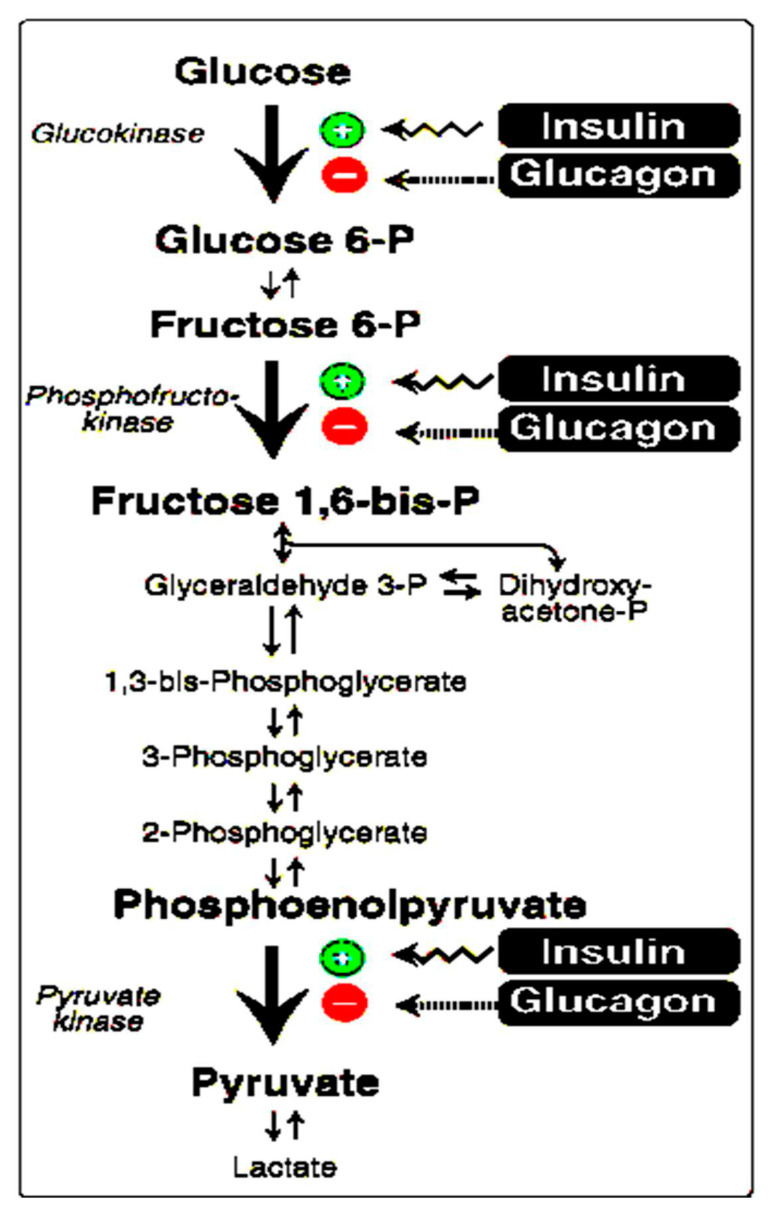
Insulin and Glucagon Regulation of Glycolytic Rate-Limiting Enzymes: Insulin stimulates the three rate-limiting enzymes of glycolysis (glucokinase, phosphofructokinase-1, and pyruvate kinase) by increasing IC pH, as these enzymes have decreased activity under acidic conditions. Glucagon opposes insulin’s action. Adapted from Lippincott’s Illustrated Reviews: Biochemistry (online version).

**B. Metabolic/molecular effects.**

i. Effects of pH on enzyme activities and macromolecule synthesis: Various environmental factors, including pH, can affect the rate of enzyme-catalyzed reactions through reversible or irreversible changes in protein structure. Most enzymes have an optimum pH at which the velocity of the catalyzed reaction is maximal [76]. Intracellular protein enzymes that control cellular function and metabolism are pH-sensitive. Synthesis of DNA and RNA (Figure 1c,d) increases with increasing pHi within the physiological range, with optimum pH for DNA polymerase activity increasing with rising pH from 7.0 to 8.0 [77], probably due to the rise in free energy of ATP hydrolysis, which increases at high pH [78]. Similarly, protein synthesis (Figure 1d) is affected by pHi. In vitro studies showed that the optimum rate of protein synthesis occurs at pH 7.4 [79]. Moreover, insulin stimulates key enzymes of glycolysis (Figure 2) by increasing local pH through activation of the electroneutral NHE in the plasma membrane [80]. The activity of a rate-limiting enzyme in glycolysis, phosphofructokinase-1, increases markedly with increasing pH over the physiological range [81]. Generally, the metabolic activities of cells increase with increasing pHi within the physiological range. Hence, we expect that decreased pHi in T2D leads to a decline in macromolecule synthesis, including proteins (enzymes, hormones, and structural proteins, such as collagen and muscle proteins), DNA, and RNA, with an overall decrease in anabolism compared to catabolism [82,83]. pHi in T2D varies among cells. Importantly, pHi of pancreatic β-cells increases [38], which could explain the increase in insulin production in the early stage of the disease in response to IR. However, the impaired insulin synthesis and secretion that occurs with disease progression could be due to increased β-cell apoptosis. On the contrary, pHi of skeletal muscles declines [82], which could explain the reported decrease in protein synthesis and associated muscle atrophy in T2D [83].

ii. Effects of pH on cellular ion conductivity: Some ion channels have pH-dependent conductivity, in particular K^+^ channels in excitable cells [84]. Intracellular acidification blocks K^+^ channels, decreasing K^+^ conductance [85], hence maintaining the resting membrane potential at a more depolarized state that facilitates the occurrence of action potentials [86]. In nondiabetics, glucose-induced electrical activity of the pancreatic β-cell plasma membrane is modulated by pHi through the effect on K^+^ channels [87]. When K^+^-channel conductance decreases, the membrane is depolarized, and voltage-gated Ca^2+^ channels and Na^+^ channels are activated [88]. Intracellular increase of Ca^2+^ stimulates exocytosis of insulin-containing vesicles (Figure 1e). Therefore, pH changes indirectly regulate insulin release. The early surge of insulin secretion in T2D due IR cannot be explained in terms of CMAD and ion conductivity, since pancreatic β-cells have increased pHi, which might explain the decrease in insulin secretion as the disease progresses. In addition to K^+^ and Ca^2+^, the conductance of most, if not all, ions, including HCO_3_^−^, H^+^, Cl^−^, and Na^+^, is pH dependent, as mentioned before [3].

## 3. Local Effects of Acidosis on Individual Tissues/Organs

**Figure 3:**
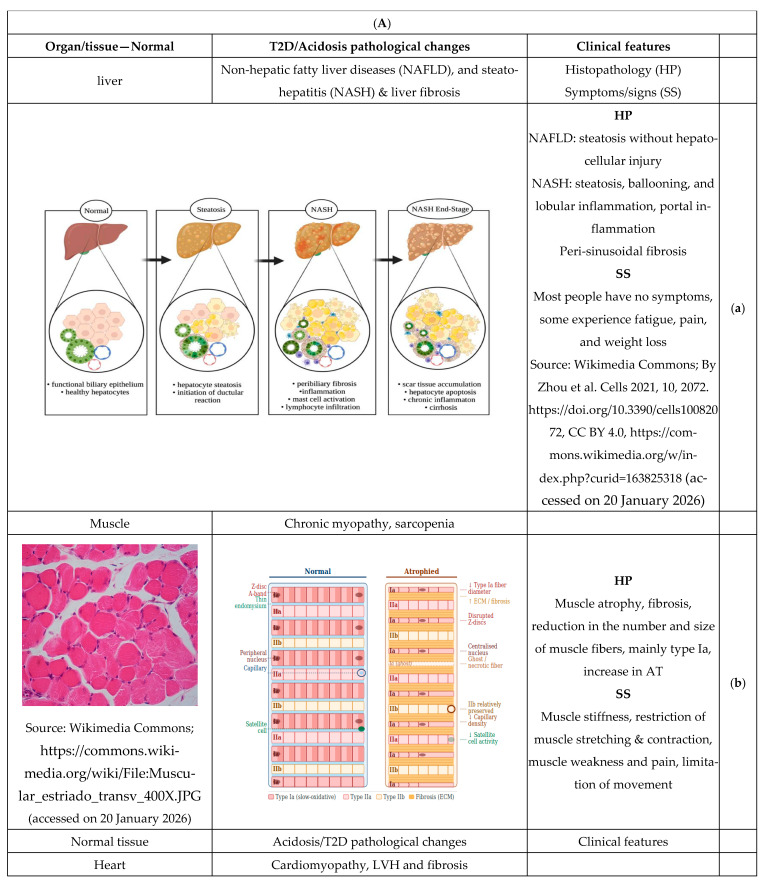

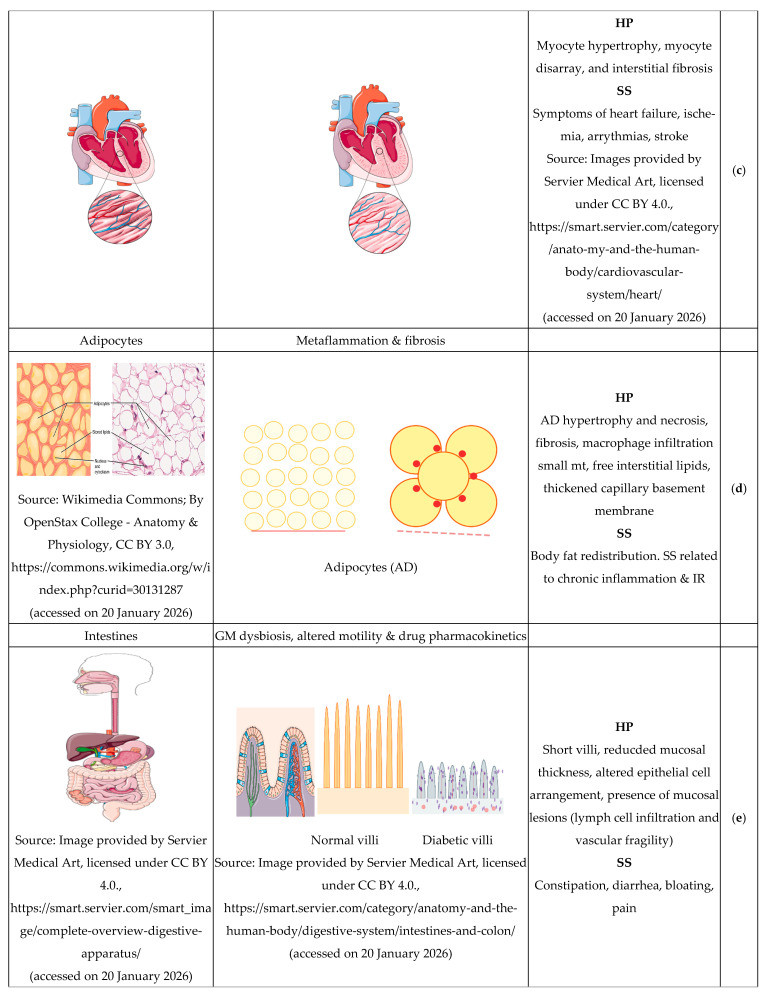

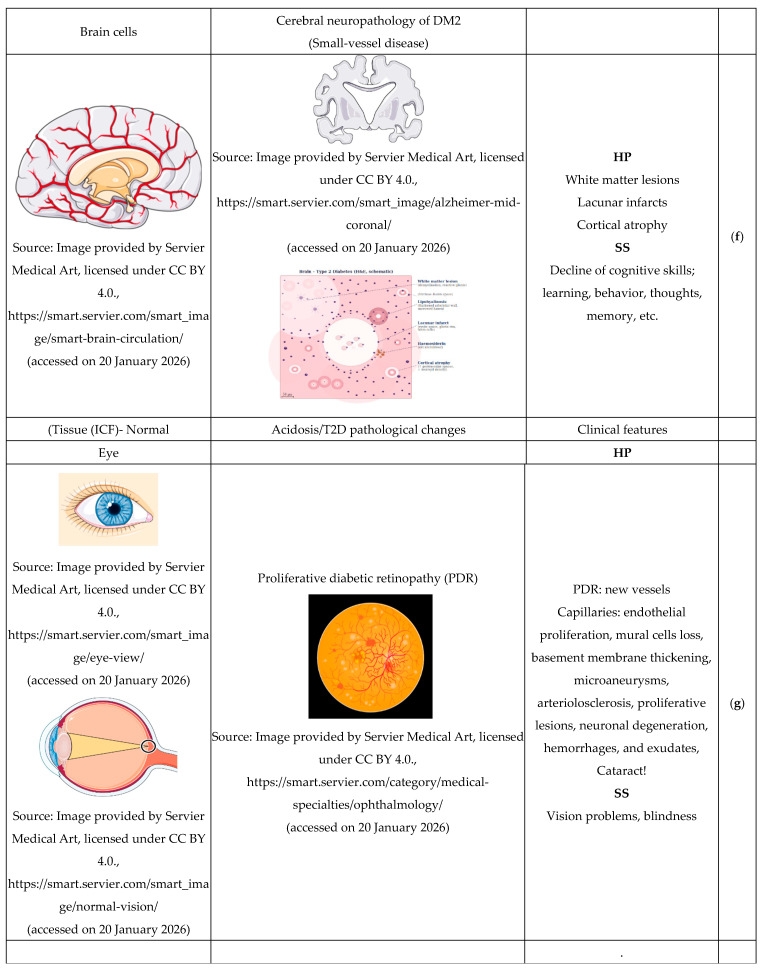

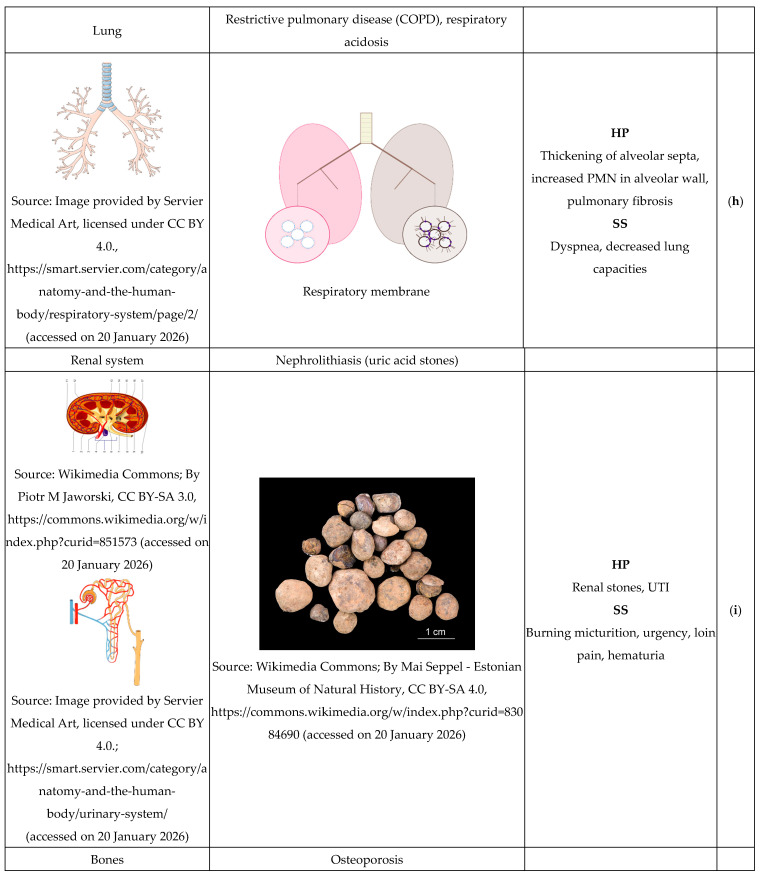

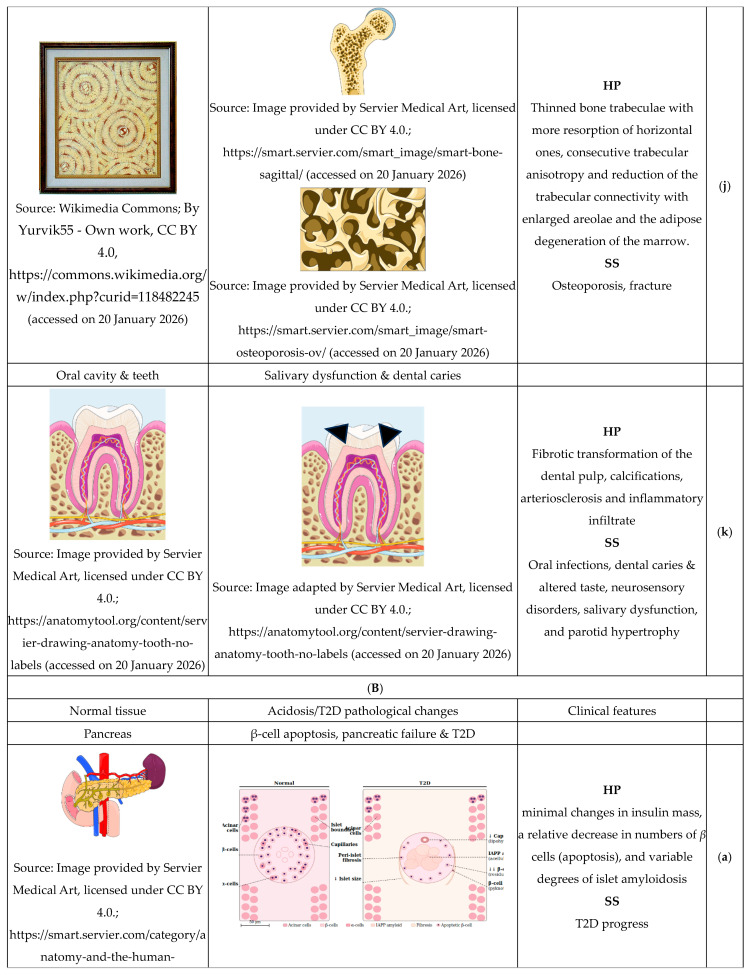

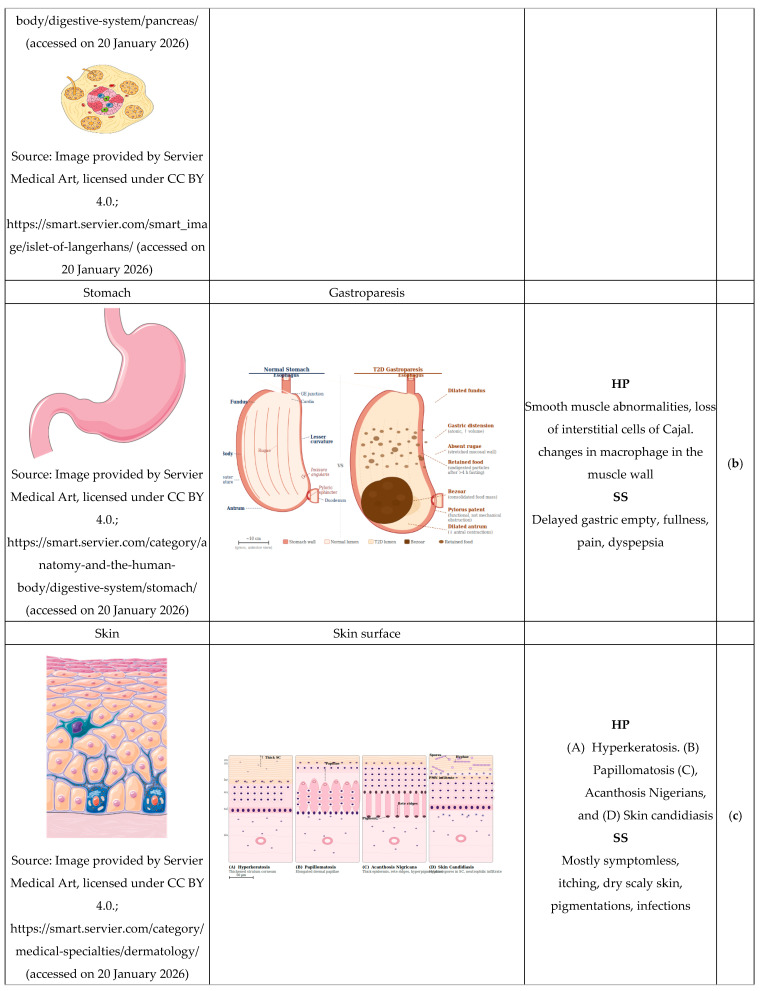
(**A**) Cluster of diagrams showing the overlapping effects of T2D and chronically increased acidity (low pH) on different organs/tissues, together with the major pathological and histopatho-logical changes, and clinical outcome. (**B**) Diagrams showing the effects of T2D and chronically decreased acidity (high pH) on different organs/tissues, together with the pathological changes, and clinical outcome. Note: In addition to the figures developed by the authors, the sources of the other figures are 1. Servier Medical Art (SMART): These are available under a CC BY 4.0 license. 2. Wikimedia Commons; https://commons.wikimedia.org/wiki/Main_Page. The links of the individual photos/figures/diagrams are attached to each figure separately, all are (accessed on 20 January 2026).

The normal pH of organs/systems, and the effects of CMA on each organ, will be discussed (Figure 3A(a)–(k),B(a)–(c)), with reference to the likely diabetic pathological/histopathological changes attributed to CMAD. Tissue pH refers only to parenchymal cells, excluding connective tissue, vascular cells, and immune cells. Furthermore, it is important to differentiate between the pHi and pHn of the specific tissue, which is vaguely distinguished in some reviewed articles.

i. Pancreas: Apoptosis of pancreatic β-cells in T2D is attributed to several factors, including oxidative stress [89], mt-dysfunction [50], and inflammation [90], all of which are associated with low pH. Metabolic stress in T2D induces pro-inflammatory cytokines, including tumor necrosis factor-α (TNF-α) and interleukin-6 (IL-6), triggering a series of complex processes that contribute to both acidosis and apoptosis in diabetic rat models [91]. Conversely, in T2D the β-cell pHi is more alkaline than normal (Figure 3B(a)), which is likely coupled with increased ECF acidity (low pHn). We speculate that low pHn and high pHi together underlie β-cell apoptosis. The pancreatic pHn has not been reported before; however, we presume it would be more acidic due to increased metabolic stress and mt-dysfunction IC, which are associated with increased proton transport to the ECF. Other contributory factors to β-cell apoptosis include glucotoxicity, lipotoxicity [92], endoplasmic reticulum (ER) stress [93], and islet amyloid polypeptide (IAPP) [94]. On the contrary, the increased β-cell pHi is a survival advantage for β-cells at the beginning of the disease. Recent research indicates that increased B-cell pHi under interstitial (pHn) acidosis contributes to β-cell dysfunction through a complex interplay among metabolic changes, altered excitability, and ion channel dysfunction, as shown in a mouse model [95]. Together, these metabolic and pH disturbances contribute to β-cell apoptosis and pancreatic failure seen in advanced T2D.

ii. Liver: The normal liver pHi was found to be 6.99 ± 0.03 (SE) in one study, and similar results have been reported, with consensus that human hepatocyte pHi is 7.0 [16,96]. Interestingly, hepatocyte pHi did not differ significantly from the normal value in acute metabolic acidosis (pH 7.04 ± 0.04) and alkalosis (pH 6.92 ± 0.08), nor during acute respiratory acidosis (pH 6.98 ± 0.04) and alkalosis (pH 7.00 ± 0.10), i.e., the liver pHi remained normal as demonstrated in vivo in dogs [37]. Hepatocyte pHi influences various hepatic functions, including urea and glucose synthesis (in suspensions of isolated rat hepatocytes) [97], biliary HCO_3_^−^ secretion (in rats) [98], and possibly mitogenesis (in cultured cells) [99]. A fall of only 0.10 pH unit inhibits glucose synthesis by 70% and urea synthesis by 50% [97]. Therefore, hepatocyte pHi is tightly maintained within the normal reference range. As reviewed by Fitz et al. [100], there are distinct mechanisms for plasma membrane H^+^/HCO_3_^−^ transport by hepatocytes, as shown in isolated rat hepatocytes. The NHE on the basolateral membrane (in contact with sinusoidal blood) contributes to recovery from IC acid load [98], and Cl^−^/HCO_3_^−^ exchange (anion exchangers—AE) on the apical membrane (in contact with the bile canaliculus) mediates HCO_3_^−^ efflux and recovery from IC alkaline load [101] in rats. While the Na^+^/HCO_3_^−^ co-transporters (NBC) are bidirectional, i.e., acid extruders and loaders, the AE functions as a “pure” acid loader. Other regulators of hepatocyte pHi are the H^+^-ATPases and K^+^/H^+^-ATPases, which use ATP hydrolysis as an energy source, or passively couple H^+^/HCO_3_^−^ movement to Na^+^/Cl^−^ movement along their electrochemical gradients [98,99]. In hepatocytes, regulation of pHi is closely linked to other cellular functions, e.g., glycolysis, cell volume control, maintenance of IC calcium, IC electrical potential, and cAMP levels, and the cellular response to hormones and growth factors [97,98,99,100,101].

Non-alcoholic steatohepatitis (NASH) and liver fibrosis are frequently associated with T2D [102,103] (Figure 3A(a)). Normally, the liver can overcome acute stress and regenerate when required; however, constant exposure to hyperglycemia, excessive lipid accumulation, and IR induces a chronic inflammatory state via lipotoxicity and oxidative stress, leading to acidosis (low pHi) and NASH. The latter is irreversible and can progress into fibrosis and eventually cirrhosis if not well managed [104]. Hepatic stellate cells play an important role in the development of NASH and liver fibrosis [105]. When stimulated by inflammatory cytokines or growth factors such as TGFβ1, stellate cells switch from epithelial to mesenchymal cells, gain fibroblastic features, proliferate, and increase the production of EC matrix components such as collagen, contributing to liver stiffness and fibrosis [105]. The role of pHi in stellate cell activation and in NASH and fibrosis is expected but has not been tested.

Exposure of isolated rat liver tissue to insulin results in increased hepatocyte pHi [106]; therefore, IR should logically result in decreased pHi. Nevertheless, even marginal increases in acidity altered the structure of macromolecules in hepatocytes [104]. The effects of low pHi in hepatocytes are similar to the effects of T2D on liver structure and function, e.g., altered lipid metabolism, fatty liver, and cellular apoptosis (Figure 3A(a)).

iii. Striated muscles: As seen in Table 2, normal human striated muscle pHi is 7.00 ± 0.06, while pHn is 7.4 [39]. In patients with T2D, skeletal muscle hexokinase II (HKII) gene expression is particularly low [107], and HKII responses to insulin are blunted [108]. HKII is the first rate-limiting enzyme in the glycolytic pathway (Figure 2). Therefore, glucose phosphorylation is diminished in the muscles of diabetic patients [109]. Although muscle phosphofructokinase-1 (PFKM) (the second rate-limiting enzyme in glycolysis) expression is increased in T2D patients, its activity decreases significantly at lower pH in frog muscles [110]. Moreover, pyruvate dehydrogenase complex (PDC) activity is decreased in patients with T2D [111]. Altogether, changes in the glycolytic pathway (Figure 2) divert pyruvate utilization from the oxidative mt pathway to the anaerobic lactate pathway, thus increasing acid formation, with a fourfold increase in lactate within skeletal muscle in T2D patients [112], and a decrease in pHi, which further decreases the pathway’s enzymatic activity. In addition, maintenance of proper insulin responses of the IRS-PI3K-AKT signaling pathway within skeletal muscles is central to maintaining glucose homeostasis. However, this pathway is impaired in the muscles of patients with diabetes [113]. This signal transduction cascade is influenced by conditions such as inflammation, oxidative stress [114], and autophagy [115], all of which are associated with CMA and T2D.

Another contributing factor to decreased muscle pHi is histidyl dipeptide buffer depletion in T2D. Histidyl dipeptides have a pK_a_ value close to physiological pH (6.8–7.1), compared with bicarbonate (pK_a_ 6.3), inorganic phosphate (pK_a_ 7.2), and histidine (pK_a_ 6.2) [116], which renders them ideal IC buffers within the pH range of skeletal muscles. Skeletal muscles are the largest reservoir of histidyl dipeptides [117], contributing approximately 7–40% of muscle buffering capacity [117,118]. Among naturally occurring histidyl dipeptides, carnosine (β-alanine-L-histidine) is the most common dipeptide present in human skeletal muscles, whereas anserine (β-alanine 3-methyl-L-histidine) and balenine (β-alanine 1-methyl-L-histidine) are largely found in other mammalian and avian species [117]. Since lactate is released from the dissociation of lactic acid with H^+^ ions, and the interconversion of pyruvate to lactate is high in diabetic muscles, an ultimate drop in muscle pHi is expected. Additionally, the reported decrease in H^+^ ion transporter expression further contributes to the muscle pHi drop in T2D [119]. In vivo and in vitro studies indicate that changes in pHi contribute to IR. The mechanisms by which pHi decline contribute to decreased insulin responses are unclear. On the contrary, IR was shown to decrease muscle pHi [120]. Understanding these findings could help develop etiology-based countermeasures to improve insulin responses in diabetic muscles.

At the molecular level, a mild decrease in pH reduces the contractility of purified actin/myosin from Dictyostelium discoideum [121], while the microtubule disassembly of bovine brain microtubule protein, which aids mitosis, increases at alkaline pH [122]. Moreover, it is known that acidification dramatically reduces the contractility of striated muscles; thus, the chronic drop of both skeletal and cardiac muscles pHi could explain the reported chronic myopathy [123] and cardiomyopathy [124] in T2D patients (Figure 3A(b,c)). Based on the above reports, which showed the bidirectional association between muscle acidosis and T2D, it is conceivable to appreciate the potential role of CMAD in T2D pathophysiology in terms of muscle atrophy and IR, and vice versa.

iv. Adipose tissue (AT): Unexpectedly, we failed to find any mention of normal human AT pHi and pHn values in the published data. AT parenchymal cells, known as adipocytes (Figure 3A(d)), spread all over the body with different types and features that work differently to regulate metabolic activities. While brown adipose tissue (BAT) has a thermogenic role, white adipose tissue (WAT) is for fat storage. WAT has two subtypes: visceral WAT (vWAT) and subcutaneous WAT (scWAT). In metabolic disorders, vWAT secretes IL-6 and plasminogen activator inhibitor-1 (PAI-1) into the portal system. On the other hand, scWAT expresses leptin and adiponectin and secretes them into the systemic circulation, contributing to metabolic homeostasis [125]. In diabetics, adipocyte mt dysfunction leads to oxidative stress and ER stress, hypoxia, and fibrosis, with consequent release of various cytokines (e.g., IL-1, IL-12) and chemokines (e.g., IL-8), which attract additional immune cells to peripheral tissues, thereby augmenting tissue inflammation. The released proinflammatory mediators (e.g., TNFα and IL-6) disrupt tissue function and induce a state of chronic, low-grade inflammation, known as metaflammation. Of note, inflammation is always associated with metabolic acidosis [126]. In WAT, pHi changes in adipocytes correlate with the movement of free fatty acids (FFA) across the cell membrane. Exposure of adipocytes to lipolytic agents or external FFAs results in rapid IC acidification that is reversed by the metabolism of the FFA or its removal by albumin. In contrast, insulin causes an IC alkalinization of AT cells. This effect is likely mediated by NHE activation, which promotes FFA esterification [127], as shown in a study of the mouse embryonic fibroblast cell line 3T3-L1. Alternatively, the effect of insulin on pHi could also arise from the removal of cytosolic FFA by esterification. Thus, the physiological response of fat cells to store and release FFA is accompanied by changes in pHi, which could also influence the subsequent signaling or hormonal effects on the cell [11]. Although there was no mention of diabetic adipocyte pHi in the literature, studies linked insulin action in AT with increased pHi, as mentioned above [125]. T2D is characterized by IR, specifically in AT [127], and IR is known to be associated with chronic acidosis. Therefore, it is acceptable to declare adipocyte pHi decline in T2D, as for the liver and muscles. However, whether that is inclusive of all body fat or limited to certain AT types is not known, although it is expected.

v. Gastrointestinal tract (Gut): The normal pH distribution of the human gut lumen follows a particular trend, with the lowest in the stomach (pH 1–3) and then increasing from the duodenum (pH 1.7–5 at proximal duodenum and pH 5–6 at distal duodenum) to the terminal ileum (pH 7–9), then dropping to 5.7 in the caecum, and increasing gradually in the ascending (pH 5.4 to 5.9) and transverse (pH 6.2) colons, the descending and rectosigmoid colons (pH 6.6 to 6.9), and reaching pH 6.7 in the rectum [27,128]. In T2D, the pH of fluids secreted from glands and gastrointestinal fluids in general is not directly influenced by the high production of H^+^ due to mt dysfunction or increased anaerobic glycolysis, since H^+^ produced in gut-lining cells due to these two conditions passes to the surrounding ISF across the basolateral membrane but not into the luminal space across the apical membrane via H^+^ transporters, such as NHE and H^+^-ATPase. However, it has been mentioned that in T2D, gastric acid secretion is significantly reduced [26], which is more pronounced in cases of diabetic patients’ gastroparesis (marked delayed gastric emptying) [129]. Furthermore, gastric pH was found to be increased in the fasting state in diabetic patients when compared with healthy individuals [130]. In T2D, decreased pHi of gastric epithelial lining cells is likely to interfere with ion conductance, specifically H^+^ extrusion, leading to IC H^+^ retention (decreased pHi) and lowered gastric fluid acidity (increased pH).

The alteration of gut pH affects: (a) gut microbiota (GM) [131], (b) gut motility [132], and (c) drug pharmacokinetics [130] (Figure 3A(e)). Similarly, T2D is also known to affect GM, gut motility, and drug metabolism [133]. T2D and gut pH, GM, and motility, individually and together, affect drug absorption and pharmacokinetics, as will be shown below. Notably, the prevalence of motility disorders is exceedingly common in diabetic patients [134], and abnormal motility of the gut, such as decreased or increased transit time, can be an independent factor affecting the amount, composition, and function of GM [135]. Furthermore, GM dysbiosis affects gut function through the ‘brain-gut axis’ by modulating afferent sensory nerves that modulate the motility of the intestine [136].

(a) Gut luminal pH and gut microbiota/microbiome: The microbiome includes bacteria, bacteriophages, fungi, protozoa, and viruses that inhabit the gut, collectively known as the microbiota. Although commonly used interchangeably, ‘microbiota’ refers to a population of microbes, and ‘microbiome’ encompasses their genomes. Gut pH alteration is known to affect gut microbiota (GM) [131,136] as shown in Fecal Enrichment Cultures and experimental animals, respectively. Equally, some clues suggest that GM contributes to CMAD, IR, and T2D development and progression [3]. The relationship between GM and CMAD is further discussed under the subtitle “The gut luminal pH and drug absorption”.
(b) Gut luminal pH and gut motility: It was previously shown that hyperosmolar solutions and HCl induce more frequent and large amplitude segmental gut contractions, whereas lipid and bile induce fewer and smaller amplitude contractions in humans [137]. The compartmentalization strategy of the esophagogastroduodenal region is to restrict high acid content to the stomach and to control acid passage to the duodenum via the lower esophageal sphincter (LES) and pyloric sphincters [138]. Both sphincters are under the control of neural reflexes involving acid-sensitive neurons that adjust the tone of the sphincters to balance the levels of acid present in the esophagus, stomach, and duodenum with the mucosal defense in these compartments [138,139]. If excessive gastric acid enters the duodenum, a duodenopylorogastric reflex is elicited, leading to contraction of the pylorus and inhibition of gastric motility, an effect that halts further gastric emptying. These coordinated motor reactions are governed by acid-sensitive neurons that, in turn, activate multiple neural circuits involving enteric, sympathetic, and vagal nerve pathways [139]. The activity of the LES is determined by two different motor programs initiated by the presence of acid in the esophagus. The first promotes esophageal peristalsis and transport of its content towards the stomach, and the second program leads to relaxation of the LES [139]. The association between delayed gastric emptying (GE) and diabetes has been known for almost a century [140]. It has been reported that 28–65% of diabetic patients experience delayed GE, which leads to a 300% longer gastric transit time in diabetic patients when compared with healthy subjects [141] (Figure 3B(b)). The significant changes in gastric motility and gastric transit time can impact the extent of absorption of diet and orally administered drugs [141]. Other changes in gut motility can lead to constipation, diarrhea, bloating, and abdominal pains (Figure 3A(e)). The positive responses of these symptoms to proton pump inhibitors (PPIs) suggest a possible major role for CMAD in gut motility disturbances in T2D.
(c) Gut luminal pH and drug absorption: As mentioned before, T2D alters gut pH and consequently affects drug absorption. Changes in aboral pH can impair the disintegration and dissolution of coated drugs, particularly those incorporating pH-sensitive materials. However, modern enteric coating materials are insoluble at normal gastric pH but begin to dissolve rapidly above a pH of 5 [142]. Gut pH and drug pK_a_ (dissociation constant) define the ionization of a drug in the human body, and the Henderson-Hasselbalch equation forms the basis for the impact of pH-pK_a_ on drug ionization and its absorption, distribution, metabolism, excretion, and toxicity (ADMET) [16]. Upon administration of a weak base, an acidic stomach contributes to its solubilization and supersaturation upon gut transfer, generating a higher compound concentration gradient, absorption, and exposure throughout the gut. However, raised stomach pH in humans due to T2D or proton pump inhibitor (PPI) intake lowers drug solubilization and supersaturation upon gut transfer and decreases downstream absorption [143].

On the other hand, the interrelation between T2D and gut acidity and motility is reflected in gut microbiota (GM) composition/function with consequent effects on drug pharmacokinetics. Microbiome composition is almost a fingerprint for each individual, and it is likely that microbiota-mediated drug metabolism also varies between individuals [144]. At the genomic level, over 270 drugs were shown to be susceptible to direct metabolism by gut bacteria, yielding inactive, more active, or even toxic metabolites [145]. Intestinal microbiota alters drug pharmacokinetics regardless of the route of administration [146]. While direct alteration of drug structure by bacterial enzymes is the common mode of microbiome-mediated drug metabolism, hepatic drug metabolism can be indirectly altered by intestinal microbiota [147], as demonstrated in germ-free mice. Metabolites produced by GM can diffuse across the gut epithelium and reach the liver via the portal vein, altering the hepatic transcriptome and thus the expression of CYP450 enzymes and drug transporters in mice [148]. GM also indirectly affects the absorption and pharmacokinetics of drugs through its impact on key local factors, such as gut pH and motility in humans [149], as part of the crosstalk. Thus, gut pH in T2D (CMAD) can affect drug efficacy in different ways. In diabetic patients, drug dosage might need to be adjusted or modified to achieve optimum efficacy. However, there is insufficient published data on this topic.

vi. Nervous system: Knowing the pHi regulation of the nervous system is central for understanding both physiological and pathophysiological changes in several neurological disorders, which are mostly associated with altered neuronal excitability. Neuronal excitability is sensitive to changes in pHi and extracellular pH due to the pH-sensitivity of different IC and EC membrane proteins, such as ion channels [150], ion transporters [151], ATPase pumps [152], and receptors [153]. Together, these proteins determine the neuron’s resting membrane potential, with consequent setting of the threshold for action potential, its duration and amplitude, and thereafter the length of the refractory period. Extracellular pH, including cerebrospinal fluid (CSF) pH and pHn in the central nervous system (CNS), is basically controlled by glial and choroid plexus epithelial cells [154]. In addition to the effects of pH on neuronal excitability, it affects the synchronization of the neuronal network and the neuron’s responsiveness to neurotransmitters. These properties permit neurons to communicate with each other and with glial cells within the CNS, supporting cognitive and other vital functions, including conscious thinking, memory, learning, behavior, and unconscious homeostatic regulation (Figure 3A(f)), as well as affecting motor control and endocrine regulation [155]. Moreover, pH influences the effects of agonist and antagonist drugs on the nervous system. In the CNS, changes in pHn are claimed to be involved in increased neuronal excitability and firing associated with seizures [156]. Acidity does not consistently result in increased neuronal excitability, as some neuronal cells exhibit reduced excitability with increased acidity, such as hippocampal neurons [155]. T2D is associated with cognitive deficit, which is reflected in accelerated decline of learning, memory, and information processing speed in humans [157], and an increased risk of dementia and Alzheimer’s disease (AD) [158]. CMAD is a likely contributor to the disturbances of these functions in diabetics. Furthermore, the main clinical manifestations of some neurodegenerative diseases include signs of increased neuronal acidity, including Alzheimer’s disease [159], Parkinson’s disease [160], and multiple sclerosis [161]. Also, acidosis was found to play an important role in the development of vascular dementia [162]. Interestingly, Alzheimer’s [163] and Parkinson’s [164] diseases are more common in T2D and share common pathophysiological features, mainly glucose hypometabolism, mt dysfunction, and oxidative stress [165], which are typical features of CMAD. Although the topic is exciting, there is insufficient data about CNS pH changes in T2D.

vii. Eyes: Studies showed that lactate increases and Ca^2+^ decreases in the vitreous body in diabetic eyes, especially in patients with proliferative diabetic retinopathy (PDR) (Figure 3A(g)). Although lactic acid production increases, pH remains nearly constant, suggesting that the human vitreous body has a robust buffering system [20]. In contrast, in diabetes, advanced glycation end products (AGEs) induce retinal neurons to reduce their pHi and increase ROS production, which leads to apoptosis and consequent PDR [166]. Altogether, these findings point to CMAD as a partner in PDR development. Although the nature of chemical changes in eye lenses suggests a possible role of pH change in cataract development, there is no epidemiological data about the frequency of cataracts in T2D, nor experimental studies exploring the effect of acidity on eye lenses or aqueous humor. Moreover, the remittent pattern of blurring of vision in diabetics raises the possibility of CMAD as a contributory factor. Finally, carbonic anhydrase (CA) blockers used in retinopathy further support the role of CMAD in diabetic eye pathology [54].

viii. Lungs: T2D is associated with impaired pulmonary function, as previously reported [167]. The typical diabetic lung is characterized by a restrictive pattern of chronic obstructive pulmonary disease (COPD) attributed to fibrosis (Figure 3A(h)). Oxidative stress and inflammation are likely involved in lung fibrosis, as shown in an experimental type 1 diabetic mouse model [168] by increasing plasminogen activator inhibitor-1 and impairing coagulation and fibrinolytic systems [169]. Underlying risk factors include hyperglycemia, hyperinsulinemia, oxidative stress, inflammation, respiratory muscle malfunction, autonomic neuropathy, glycosylation of collagen and elastin tissue proteins with alteration of connective tissue (CT), surfactant dysfunction, and micro/macroangiopathy of alveolar capillaries and pulmonary arterioles [169]. Although no study has tested the contribution of CMAD in diabetic lung, at least the first five risk factors are associated with CMAD. Thickening of the respiratory membrane and COPD result in CO_2_ retention in T2D, with consequent chronic mild respiratory acidosis, adding to CMAD. There is no data about lung parenchyma pHi and pHn or alveolar lining fluid pH. We speculate that in T2D, lung pH is low.

ix. Urinary system: Reduced urine pH in T2D has been shown in different studies. A link between low urine pH and IR and obesity, which are both associated with T2D, was previously reported [170]. Also, patients with metabolic syndrome were shown to have significantly low urine pH and 24 h urine pH [171]. On the other hand, T2D is known to be associated with an increased risk for nephrolithiasis, particularly uric acid (UA) stones (Figure 3A(i)) [172]. Urine pH is a key determinant of UA solubility, and markedly acidic urine is recognized as a risk factor for UA crystallization and stone formation. Moreover, unduly acidic urine (pH < 5.5) is frequently recognized in T2D patients with [172] and without UA stones [173]. Overly acidic urine, which predisposes diabetic patients to UA stone formation, persists even after correction for confounding factors, such as diet, body size, age, renal function, and ethnicity [30]. While acidic urine is one of the features of CMAD and a contributor to increased susceptibility to renal stones in diabetic patients, diabetic nephropathy contributes to CMAD development since the kidney is the key organ in acid–base homeostasis. The kidney is the main exit site for acid from the body and an important source of HCO_3_^−^ as a buffer. Moreover, it controls the excretion of ammonia and urea in response to the body’s acid–base status. Finally, pHi and extracellular pH in different parts of the nephrons/kidney are a top priority for understanding the contribution of nephropathy to CMAD, and vice versa. However, this is a large and complex topic that is beyond the scope of this review.

x. Skin: The skin surface acts as a permeability barrier and a cutaneous antimicrobial defense, with a surface pH (the acid mantle of the stratum corneum) of <5.0 and a mean sweat pH of 6.3 [174]. In T2D, the skin is more alkaline than in healthy subjects (Figure 3B(c)), unlike most other tissues (Figure 3A). The pH of the skin follows a sharp gradient of increasing acidity across the stratum corneum from deep inside to the surface, as a requirement for control of enzymatic activities and the skin wear and tear phenomenon. It is estimated that the ‘natural’ skin surface pH is on average 4.7 [175] and that it varies considerably from 4.0 to 7.0, based on site and other factors. Skin with pH values < 5.0 is superior to skin with pH values > 5.0 in terms of biophysical barrier function. Skin acidity is attributed to proteins of the stratum corneum and amino acids of sweat, sebum, and lactic acid [176], and it is built by passive and active processes involving eccrine and sebaceous secretions and proton pumps. It is affected by endogenous factors, including skin moisture, sweat, sebum, anatomic site, age, genetics, ethnicity, and sex, and exogenous factors, including soaps, cosmetics, dressings, humidity, and antibiotics [177,178]. Skin pH is higher in women [176], and it varies between different body areas. For example, it is higher in the urogenital folds and apocrine glands [178].

Changes in pH are reported to play a role in the pathogenesis of skin abnormalities in different diseases, including T2D. An acidic skin pH (4–4.5) was found to keep resident bacterial flora attached to the skin, whereas an alkaline pH (8–9) promotes its dispersal from the skin. In T2D patients, skin pH is a risk factor for skin candidiasis [179]. In one study, T2D patients were found to have significantly higher pH in intertriginous areas, with a higher risk of intertrigo Candida infection [180]. In an in vitro study, the hypha form, which grows optimally at a pH > 6.5, is the initial invader into host tissue [179]. Moreover, in T2D, areas of the skin with high pH are subject to hyperkeratosis, papillomatosis, and overt acanthosis nigricans, which is due to either hyperinsulinemia or growth factors such as transforming growth factor and epidermal growth factor [180]. The possibility that CMAD could be a contributory factor is not excluded. Also, skin itching in T2D could be explained in part by changes in skin pH. However, there is only limited published data about skin pH in diabetic patients.

xi. Bones: CMAD was found to increase osteoclast activity, with consequent bone resorption and release of Ca^2+^ and anionic proton buffers in animal models [181]. Acidosis is known to induce the formation and activation of osteoclasts through stimulation of two different Transient Receptor Potential (TRP) cation channels, TRPV1 and TRPV4, as shown in vitro and in vivo in mouse studies [182,183]. TRPV1 is a polymodal receptor and a nonselective cation channel that is activated by heat, low pH, and some chemicals. Correcting local acidosis in the bone marrow (BM) has proven extremely challenging due to difficulties in reaching the BM site, whereas systemic administration of a TRPV1 antagonist to reduce osteoclast activation is clinically possible. Other studies reported that the main players involved in osteoclast differentiation and activation under physiological conditions are Receptor Activator of Nuclear Factor Kappa-B Ligand (RANKL), produced by stromal cells, and its decoy receptor, osteoprotegerin (OPG) [184]. The binding of RANKL to its receptor RANK on osteoclast precursors promotes cell differentiation through activation of Akt, NF-κB, and MAPK pathways [184]. Furthermore, hypoxia is a known inducer of genes involved in osteoclastogenesis and is thought to act through hypoxia-inducible factor-1 (HIF-1), but this mechanism remains elusive [185]. Hypoxia results in pH reduction in cell media and is one of the mechanisms involved in osteoclast activation [185].

Several diabetic complications are often overlooked, including disrupted bone homeostasis, osteoporosis, and fractures, especially hip fractures (Figure 3A(j)) [186]. Increased bone fragility is frequently linked to normal or even elevated bone mineral density in T2D [186]. The impact of high blood glucose on osteoclast activation has been demonstrated previously using RAW264.7 cells and Bone Marrow Macrophages as models [187], although a meta-analysis showed that glycated hemoglobin, a measure of glycemic control, is not associated with osteoporosis in diabetic patients [188]. However, it is confirmed that diabetics experience increased bone fragility, probably due to BM acidosis that triggers osteoclast activation by modulating TRPV1 [41]. In addition, chronic acidosis inhibits osteoblasts (bone-building cells) and reduces bone formation [181]. Interestingly, correcting BM acidosis with nanotechnology showed positive results in terms of bone health and protection against osteoporosis in limited trials [54], suggesting a possible role for CMAD on diabetic bone fractures.

xii. Saliva and teeth: The human saliva has a normal pH range of 6.2–7.6, with an average pH of 6.7 [189]. The oral cavity pH is maintained close to neutrality (6.7–7.3) by saliva through two mechanisms. First, the flow of saliva eliminates carbohydrates that could be metabolized by bacteria to produce acids. Second, the buffering activity of saliva neutralizes acidity from food and drinks, as well as from microbial activity [189]. Alterations in salivary pH are often reported in diabetic patients. Salivary pH was found to be significantly lower in diabetic patients [190], a finding supported by another study where salivary pH was 6.5 in diabetic patients compared to 7.88 in non-diabetic subjects [189]. The acidic pH of saliva in diabetics may be attributed to microbial activity or decreased levels of salivary bicarbonate associated with decreased saliva flow rate [190]. Low salivary pH provides an acidogenic environment for the growth of aciduric bacteria, leading to a vicious cycle of declining pH that aggravates dental caries. The oral manifestations of T2D include salivary dysfunction, dental caries, mucosal and other oral infections (e.g., gingivitis and periodontitis), and taste and neurosensory disorders, which are largely attributed to salivary acidity (Figure 3A(k)) [189]. Finally, acid-neutralizing therapy was shown to be successful in alleviating oral and dental diabetic symptoms, at least in one study [54]. Accordingly, CMAD is likely to be the principal contributor to dental caries and other oral manifestations in T2D.

xiii. Connective tissue (CT) and joints: The effects of acidosis on CT have been known for a long time [191]. CT is the structural component of almost all body organs, while the EC matrix (ECM) forms the main ground substance of CT with collagen and elastin. Changes in CT secondary to CMAD are likely to influence the pathophysiological changes of diabetes in all organs, specifically the locomotor system, including muscles, bones, cartilage, and joints, which are rich in CT. The major molecular components of ECM are proteoglycans, which are formed of protein and glycosaminoglycan (GAG) components with multiple charged functional groups [192]. Therefore, even slight changes in blood pH can lead to changes in the characteristics of proteoglycans. The binding of ECM proteins to GAGs is primarily electrical charge-dependent. In cartilage, proteoglycans, namely hyaluronic acid, represent a high-molecular-weight polyanionic complex that forms the important compressible component of cartilage due to their high water-binding capacity [192]. An example of a CT disorder that affects joints and is associated with both T2D [193] and CMA [194] is osteoarthritis. Acidosis of synovial fluid has been shown to correlate with radiological features of joint destruction and granulocyte build-up in the knee in rheumatoid arthritis patients [195]. It was claimed that an acidic ECF in the arthritic joint may subsequently result in increased chondrocyte IC acid load, potentially driving disease progression [196]. In chondrocytes isolated from bovine articular cartilage, minor alterations in pH impact chondrocyte metabolism and ability to synthesize GAGs, with maximum synthesis occurring at a pH of 7.2 [197]. Further evidence for the role of CMAD in joint inflammation is the positive response in patients with osteoarthritis to alkaline mineral supplements [196]. On the other hand, T2D is associated with several CT and joint diseases, including diffuse idiopathic skeletal hyperostosis, diabetic hand syndrome, limited joint mobility, Dupuytren’s disease, flexor tenosynovitis/trigger finger, carpal tunnel syndrome, adhesive capsulitis, rotator cuff tendinopathy, calcified tendinitis, Achilles tendinopathy, and Achilles tendon tear [123]. Although the above T2D-associated joint and CT diseases were not reported to be associated with CMAD, their pathological features are consistent with those seen in CMAD. Therefore, further studies are required to confirm this association.

xiv. Immune system/inflammation: Several studies have demonstrated the association between acid–base disturbance and disorders of the immune/inflammation system. The contribution of immune cells and cytokines (IL1, TNF, IL6, IL17, TLR, etc.) in association with gut microbiota and inflammation to CMAD was recently reviewed [3]. Moreover, CMAD and inflammation are in crosstalk and potentiate each other in a vicious cycle [3]. The less explored immune parameter in CMAD is nitric oxide (NO). It was shown that acidosis regulates and favors the formation of both inducible nitric oxide synthase (iNOS) and endothelial NOS (eNOS), in rat studies [198,199]. NO contributes to the local control of blood flow during hypoxia/ischemia, parallel to lactic acid production, suggesting that pH is critically involved in inflammation and endothelial dysfunction in diseases associated with CMA [200]. NO is an important part of the host defense mechanism, and it displays both pro- and anti-inflammatory properties depending on its location and concentration [201]. Excess NO increases inflammation marker levels in T2D patients via activation of the peroxisome proliferator-activated receptor gamma (PPARγ)/eNOS pathway [202]. On the contrary, excessive or inappropriate NO production can cause tissue damage. Moreover, systemic and local administration of NOS inhibitors ameliorate and exacerbate the inflammatory response, respectively, in rats [200]. Further evidence for the role of acidosis in the immune system and inflammation is the role displayed by NaHCO_3_. Interestingly, the acid-neutralizing supplement NaHCO_3_ was found to shift macrophage polarization from pro-inflammatory M1 to anti-inflammatory M2 phenotype in rats, suggesting that NaHCO_3_ reduces inflammation. The anti-inflammatory effect of NaHCO_3_ was found to be mediated through activation of the cholinergic anti-inflammatory pathway via the vagus nerve in rats [201].

**Effect of CMAD on other tissues and functions:** Indeed, several other tissues and systems are affected by T2D, and several functions are influenced negatively by CMAD since T2D is a typical systemic disease that could affect most body tissues. However, only selected tissues are reviewed here based on data availability and reliability. On the other extreme, data about an affected system or functions could be huge, e.g., the kidney, pregnancy, development, fertility, etc., and therefore will be reviewed separately. Finally, in many tissues, particularly the adipose tissue and the nervous system, direct pH measurements in T2D are lacking, and this limitation is explicitly acknowledged when interpreting the pathophysiological relevance of CMAD.

## 4. Therapeutic Potentials of pH Correction in T2D

The Supplementary Table S1 shows a list of acid-neutralizing therapies quoted from Giha et al. [28]; some are under research trials for use in T2D and/or its complications, or other similar disorders, e.g., metabolic syndrome and IR.

The therapeutic observations summarized in this section are presented solely to provide proof-of-concept support for the mechanistic role of CMAD in T2D pathophysiology. They are not intended to constitute clinical recommendations, and readers should not interpret any intervention described here as validated for use in T2D outside of investigational settings. No pH-directed intervention has to date been evaluated in an adequately powered, randomized controlled trial specifically designed to test acid–base correction as a primary therapeutic strategy in T2D. The evidence presented falls into two distinct tiers, which are maintained throughout this section.

Tier 1: Interventions with limited clinical evidence in T2D or closely related populations includes sodium bicarbonate supplementation, for which randomized controlled trials conducted in patients with chronic kidney disease and comorbid metabolic acidosis have reported improvements in insulin sensitivity and indices of metabolic function [203,204], and dietary alkalization strategies, for which observational and limited interventional data exist in metabolic syndrome cohorts [205,206]. Proton pump inhibitors are included in this tier with the explicit qualification that their use in T2D is now indicated only for gastrointestinal comorbidities [207]; any observed metabolic effects are secondary, incompletely characterized [208], and should not be interpreted as constituting a therapeutic rationale for their use as acid-neutralizing agents in T2D.

Tier 2: Experimental and preclinical observations encompass nanomedicine-based pH correction strategies [209], gene therapy approaches targeting pH-regulatory pathways [210], immune modulation via pH-sensitive mechanisms [201,202], and organ-specific buffering interventions [54]. These are research-stage observations derived from cell culture, animal models, or early-phase human studies, with no current clinical applicability in T2D. They are discussed here exclusively as mechanistic proof-of-concept observations that support the biological plausibility of the CMAD hypothesis.

Any discussion of pH-directed therapy in T2D must be accompanied by an explicit acknowledgment of the potential risks, adverse effects, and confounding factors that would complicate its clinical translation. These have not been systematically studied in T2D populations and represent important unknowns that future trials must address.

First, overcorrection of acidosis carries a genuine hazard. Excessive alkalinization, whether from sodium bicarbonate supplementation, high-dose alkaline mineral intake, or combined interventions, risks inducing metabolic alkalosis, which can paradoxically impair tissue oxygen delivery via the Bohr effect [211]. Also, overcorrection suppresses hypercapnic respiratory drive [212], precipitates hypocalcaemic tetany through pH-dependent shifts in calcium binding to albumin [213], and exacerbates hypokalaemia in T2D patients already predisposed by diuretic use, hyperaldosteronism secondary to nephropathy, or gastrointestinal losses [214]. Importantly, the therapeutic window for alkalinization in CMAD has not been defined.

Second, sodium bicarbonate supplementation imposes a sodium and fluid load that is clinically relevant in the T2D population, where hypertension, congestive heart failure, and diabetic nephropathy are prevalent comorbidities [215]. The populations in whom CMAD is most severe, those with advanced nephropathy, longstanding disease, and the greatest metabolic derangement, are frequently the same patients in whom sodium loading carries the greatest cardiovascular and renal risk [216], creating a fundamental tension in the therapeutic strategy.

Third, chronic proton pump inhibitor use is associated with hypomagnesaemia [217], vitamin B12 deficiency [218], elevated risk of Clostridioides difficile infection [219], and potential acceleration of chronic kidney disease progression [220]. All of the above are of disproportionate relevance to T2D patients and must be weighed against any putative metabolic benefit.

Fourth, and critically, T2D patients present with a complex polypharmacy profile in which many standard-of-care agents independently alter acid–base balance. Metformin reduces lactate clearance and carries a low but established risk of lactic acidosis [221]; SGLT2 inhibitors can induce euglycaemic diabetic ketoacidosis [222]; ACE inhibitors and angiotensin receptor blockers promote hyperkalaemic acidosis in the context of nephropathy [223]; and loop diuretics generate metabolic alkalosis [224]. Any future interventional trial of pH correction in T2D must rigorously control for these confounders, and any retrospective or observational interpretation of acid–base data in T2D patients receiving these agents should account for their independent effects on pH.

Fifth, systemic alkalinization cannot be assumed to produce uniform pH correction across all tissue compartments. Given the compartment-specific nature of pH dysregulation in T2D described throughout this review [3], a systemic intervention may correct acidosis in one compartment while failing to address another, or may generate adverse transmembrane pH gradients with unpredictable functional consequences [225]. Targeted, tissue-specific pH correction, while conceptually attractive, remains a technical and pharmacological challenge that has not been resolved.

## 5. Revisiting T2D Pathophysiology

This comprehensive review showed major gaps in the literature regarding the human studies of CMA in T2D, although the interpretation of the existing scattered data from different resources, some of which are from experimental and in vitro studies, shows compelling evidence that chronic metabolic acidosis of diabetes (CMAD) is a possible important, yet historically underappreciated, dimension of T2D pathophysiology.

It is essential to state at the outset that the framework developed in this review represents a hypothesis-driven conceptual synthesis and not an established causal model. The central hypothesis is that CMAD, characterized by mild, sustained reductions in intracellular and interstitial pH across multiple tissues, functions as a mechanistically active contributor to T2D pathophysiology, both driving and being driven by insulin resistance, and thereby amplifying organ dysfunction across multiple tissues simultaneously. This hypothesis is supported by a convergence of indirect lines of evidence spanning in vitro enzyme kinetics, animal models, and limited human observational data, but it has not been confirmed by prospective, interventional, or longitudinal human studies designed to test it directly. Critical limitations constrain the strength of all inferences drawn: direct measurements of tissue-specific intracellular and interstitial pH in human diabetic cohorts are scarce or absent for several metabolically central tissues, including adipose tissue, the central nervous system, and pancreatic β-cells; the mechanistic links between pH shifts and cellular dysfunction are largely derived from acute acidification experiments that may not replicate the mild, chronic nature of CMAD; and the relative quantitative contribution of CMAD to T2D pathology, compared to hyperglycemia, lipotoxicity, oxidative stress, and inflammation, remains unquantified. No pH-directed intervention has been evaluated in an adequately powered, randomized controlled trial specifically targeting T2D outcomes. This review should therefore be read as a mechanistically coherent, testable hypothesis intended to stimulate systematic investigation, and not as a basis for changes to clinical practice. Finally, it is important to emphasize that the model proposed in this review, positioning CMAD as a mechanistically active contributor to T2D pathophysiology, is largely inferential. The framework presented here is built on a convergence of indirect lines of evidence rather than a body of direct, prospective, and interventional data (Table S2). Therefore, readers should interpret the conclusions accordingly.

By systematically reporting the effects of altered pH homeostasis from molecular mechanisms through cellular processes to organ-system dysfunction, there is a striking alignment between the biological consequences of CMA and the features of T2D.

At the molecular level, pH disturbances significantly impact enzyme kinetics, macromolecule metabolism, and ion conductance, fundamental processes that govern cellular function. The pH-dependent optimization of principal glycolytic enzymes, including hexokinase II, phosphofructokinase-1, and pyruvate kinase, may link IC acidification to impaired glucose metabolism and IR. Similarly, the pH sensitivity of DNA and RNA synthesis, along with protein production, can provide a mechanistic basis for understanding the metabolic dysfunction characteristic of T2D.

At the cellular level, the pH shifts critically influence both cell cycle progression and apoptotic pathways. The requirement for alkaline pHi to transition from G0/G1 to S phase, coupled with the activation of caspases and pro-apoptotic proteins under acidic conditions of CMA, offers insight into pancreatic β-cell failure and the progressive loss of insulin secretion in T2D. The widespread apoptosis documented across multiple tissues in T2D, from pancreatic β-cells to skeletal muscle, neurons, and hepatocytes, may thus represent a unified response to chronic IC acidification rather than independent pathological processes.

Most compellingly, the systemic manifestations of CMA mirror the multi-organ pathology observed in T2D with remarkable fidelity. In the pancreas, the paradoxical IC alkalinization of β-cells against a backdrop of EC acidification may explain both the initial compensatory hyperinsulinemia and subsequent β-cell exhaustion. In skeletal muscle, acidosis-induced impairment of insulin signaling pathways and reduction in histidyl dipeptide buffering capacity directly contribute to both IR and sarcopenia. Hepatic acidification aligns with non-alcoholic steatohepatitis and liver fibrosis, while adipose tissue pH changes correlate with inflammatory mediator release and metabolic dysregulation seen in T2D.

Beyond these central metabolic tissues, CMAD can provide an explanation for numerous T2D complications previously attributed solely to hyperglycemia or microvascular disease. The association between urinary acidification and uric acid nephrolithiasis, the role of salivary pH reduction in dental caries, the contribution of bone marrow acidosis to osteoporosis and fracture risk, the impact of altered gut pH on microbiota dysbiosis and gastroparesis, and the influence of skin pH changes on infectious complications, all exemplify how CMA effects align with the protean manifestations of diabetes.

The therapeutic implications of recognizing CMAD’s possible role are notable. The demonstrated efficacy of acid-neutralization strategies, ranging from dietary modifications and alkaline supplements to proton pump inhibitors and targeted nanomedicine approaches, in relative amelioration of diabetic symptoms and slowing disease progression worth pragmatic testing and validation. The anti-inflammatory effects of sodium bicarbonate through macrophage polarization and cholinergic pathway activation, the protective effects of pH correction on bone density, and the improvement in β-cell function with buffering capacity enhancement all support the potential for pH-directed interventions in T2D management.

### 5.1. Integrative Mechanistic Framework: pH Shifts, Signaling Pathway Disruption, and Metabolic Consequences in T2D

The preceding sections of this review have described the effects of CMAD across individual tissues and molecular targets. The purpose of this subsection is to synthesize those observations into a coherent mechanistic framework that makes explicit how pH shifts initiate, propagate, and amplify the signaling disruptions that produce the metabolic consequences characteristic of T2D. To promote transparency and scientific rigor, each mechanistic link is classified throughout as **Established** (supported by direct human or ex vivo human tissue data in the relevant pH range), **Supported** (demonstrated in animal models or in vitro at pH ranges consistent with CMAD, without direct human confirmation), or **Speculative** (inferred by logical extrapolation from established pH–biology relationships in non-diabetic systems, with no confirmatory data in diabetic tissue). No pathway is presented as dominant over others unless supported by quantitative human evidence, which, as detailed in Section 5.2, remains limited for most of the mechanistic links described.

**Figure 4:**
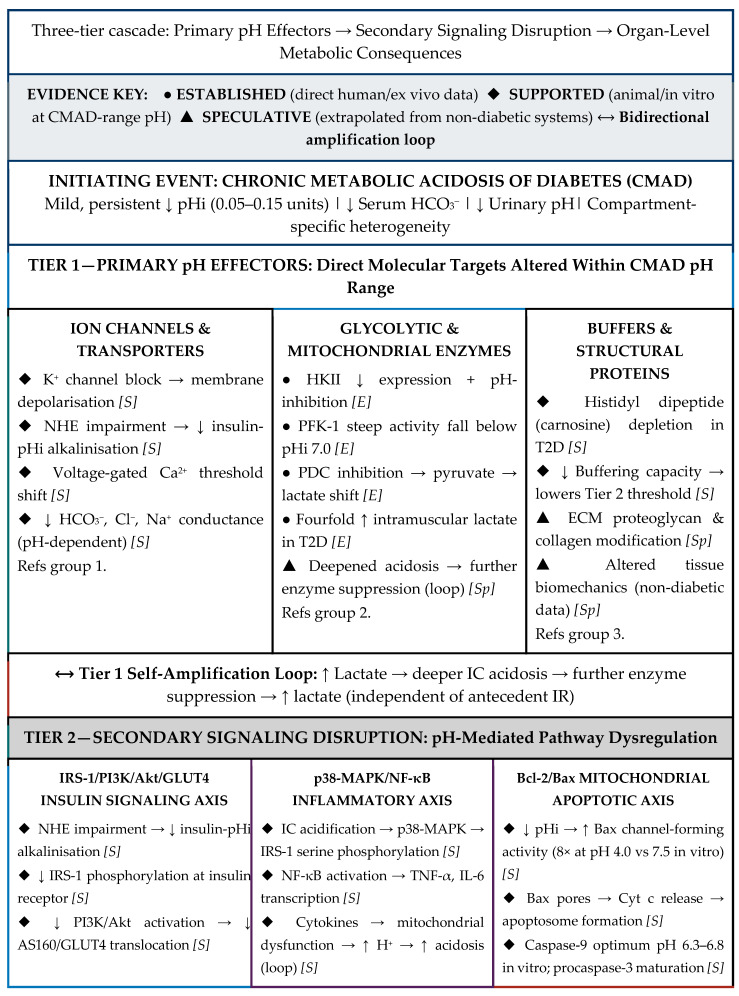

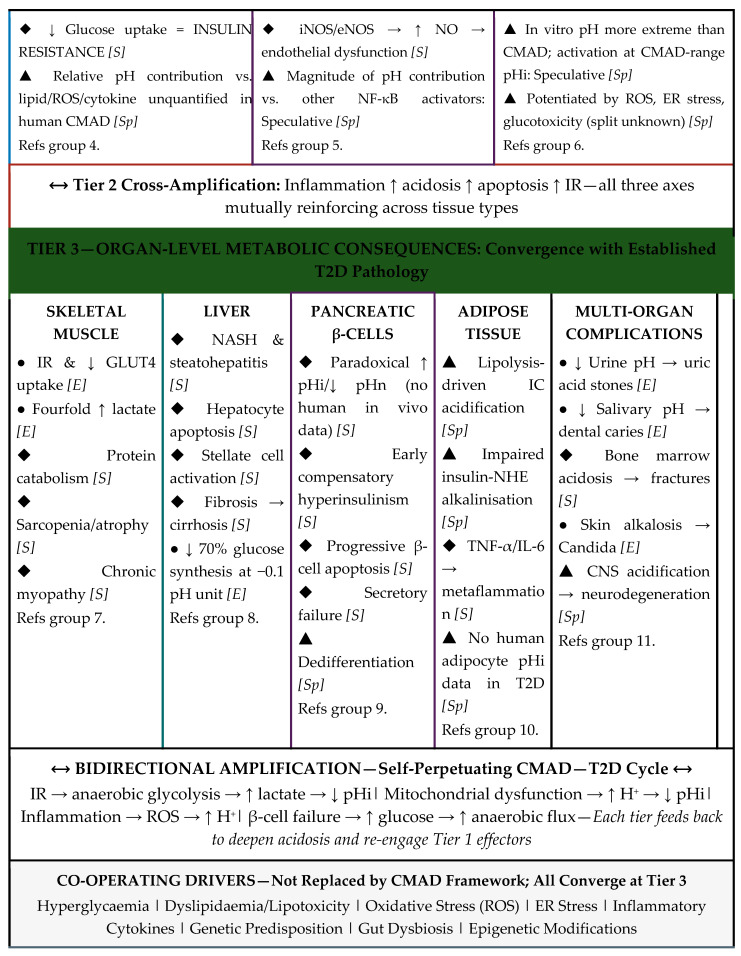

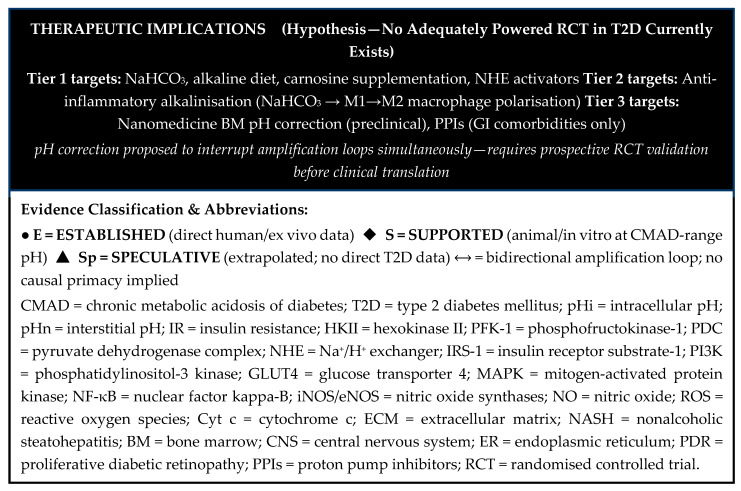
Integrative Mechanistic Framework: CMAD-Driven Signaling Disruption in T2D. References are cited in 11 groups, one group in each cell of the diagram, starting from upper left (group 1), extending to the right (horizontal): groups 1. [3,80,84,85,86,87,88], 2. [81,106,107,108,109,110,111], 3. [114,115,116,189,190], 4. [6,80,111,112], 5. [67,91,196,197,198,199,200], 6. [50,59,60,61,63,64,65,75], 7. [40,52,82,83,110,111,117,118,119,121,122]. 8. [16,95,100,101,102,103,104], 9. [53,59,87,88,91,92,93,94], 10. [123,124,125], and 11. [22,23,41,107,157,164,165,168,169,170,171,172,173,174,175,176,177,178,179,180,181,182,183,184,185,186,187].

The framework operates across three tiers, from primary molecular pH sensors through secondary signaling cascades to organ-level metabolic outcomes, with bidirectional amplification loops operating between and within tiers, as shown in (Figure 4).

**Tier 1—Primary pH Effectors: Direct Molecular Targets of Intracellular Acidosis**

The first tier comprises the molecular species whose activity is directly altered by pH shifts within the range documented in CMAD, approximately 0.05 to 0.15 pH units below normal pHi, without requiring intermediate signaling steps. Three categories of primary pH effectors are relevant to T2D pathophysiology.

The first category is pH-gated ion channels and transporters. Intracellular acidification blocks ATP-sensitive K^+^ channels in pancreatic β-cells and excitable tissues, shifting membrane potential toward depolarization and altering the threshold for voltage-gated Ca^2+^ channel activation [84,85,86] (**Supported**: demonstrated in rodent β-cells and excitable tissues; not directly confirmed in human diabetic β-cells in vivo). The NHE family of Na^+^/H^+^ exchangers, which normally mediates cytoplasmic alkalinization in response to insulin stimulation, is impaired under chronic acidotic conditions, reducing the insulin-dependent rise in pHi that is required for downstream metabolic signaling [80] (**Established** in hepatocyte and myocyte models; human data limited to indirect inference from NHE activity assays). The conductance of HCO_3_^−^, Cl^−^, and Na^+^ channels is similarly pH-dependent, collectively determining the electrochemical environment within which insulin signaling operates [3].

The second category is glycolytic and mt enzymes. Hexokinase II (HKII), the first rate-limiting enzyme of glycolysis in skeletal muscle and adipose tissue, exhibits reduced expression in T2D [107,108] (**Established** in human skeletal muscle biopsy studies), and its activity is further suppressed by the low pHi associated with CMAD [109] (**Supported**: pH–activity relationships characterized in vitro). Phosphofructokinase-1 (PFK-1), the second rate-limiting glycolytic enzyme, displays steep pH-dependence across the physiological range, with activity falling markedly as pHi declines below 7.0 [81] (**Established** in vitro; consistent with clinical observations in diabetic muscle). The pyruvate dehydrogenase complex (PDC), which gates pyruvate entry into the tricarboxylic acid cycle, is inhibited in T2D [111] (**Established** in human diabetic muscle), and its activity is further attenuated at reduced pHi (**Supported**: mechanistic inference from the pH–enzyme kinetics literature). The collective consequence of HKII, PFK-1, and PDC suppression under acidic conditions is a metabolically coherent shift: pyruvate flux is diverted from oxidative mt metabolism toward anaerobic lactate production, generating a fourfold increase in intramuscular lactate [112] (**Established** in human T2D) and a progressive deepening of IC acidosis that constitutes a self-reinforcing tier 1 loop.

The third category is structural and buffering proteins. Histidyl dipeptides, principally carnosine in human skeletal muscle, serve as the primary IC buffers within the physiological pH range (pK_a_ 6.8–7.1) and are depleted in T2D [116,117,118] (**Supported**: animal and human biopsy data; depletion rate in longitudinal human T2D cohorts not established). Their depletion reduces the muscle’s capacity to resist acidification, lowering the threshold at which tier 2 signaling disruption is engaged. Collagen and elastin within the extracellular matrix undergo structural modification at even slight pH reductions, altering their biomechanical properties and the tissue microenvironment within which cellular signaling occurs [191,192] (**Speculative** in the specific context of CMAD-driven T2D; pH–matrix relationships established in non-diabetic connective tissue studies).

**Tier 2—Secondary Signaling Disruption: pH-Mediated Pathway Dysregulation**

The second tier encompasses the downstream signaling consequences of Tier 1 dysfunction. Three principal signaling axes are affected, each with distinct metabolic and cellular outcomes, and each operating to varying degrees in parallel rather than in strict linear sequence. It is important to emphasize that the relative contribution of pH-mediated disruption to each of these pathways, compared to other established drivers such as lipid intermediates, ROS, and inflammatory cytokines, has not been quantified in human CMAD and should not be inferred from the order in which they are discussed below.

The first signaling axis is the IRS-1/PI3K/Akt/GLUT4 insulin signaling cascade, which represents the most directly pH-linked and mechanistically characterized axis in the context of CMAD. Acidosis-impaired NHE activity reduces the insulin-stimulated cytoplasmic alkalinization that normally facilitates IRS-1 phosphorylation and its recruitment to the activated insulin receptor [80] (**Supported**: NHE-pHi-insulin signaling linkage demonstrated in in vitro and rodent models). Downstream, IC acidification directly disrupts Akt phosphorylation and activity [6] (**Supported**: demonstrated in cell culture; human in vivo quantification absent), impairing the Akt-dependent phosphorylation of AS160 (TBC1D4) and the consequent translocation of GLUT4-containing vesicles to the plasma membrane [6] (**Supported**). The functional result is reduced glucose uptake in skeletal muscle and adipose tissue, the tissue-level correlate of IR. It must be noted that this axis is one of several converging mechanisms of IR in T2D, including serine phosphorylation of IRS-1 by inflammatory kinases, diacylglycerol-mediated PKC activation, and mt ROS generation, and pH-mediated disruption should not be construed as the singular or dominant mechanism of IR in the absence of human quantitative data (**Established** that the pathway is pH-sensitive; **Speculative** that pH is the primary driver of its impairment in human T2D).

The second signaling axis is the p38-MAPK and NF-κB inflammatory pathway. IC acidification activates the p38 mitogen-activated protein kinase (MAPK) stress-response pathway [226] (**Supported**: demonstrated in cell culture models at pH ranges consistent with CMAD), which mediates serine phosphorylation of IRS-1 at inhibitory residues, thereby independently impairing insulin signaling and establishing a mechanistic bridge between the metabolic and inflammatory arms of IR. Concurrently, acidosis promotes NF-κB activation [3], driving transcription of pro-inflammatory cytokines including TNF-α and IL-6 [91] (**Supported**: association between acidosis and NF-κB pathway demonstrated in inflammatory cell models; direct demonstration in human diabetic tissue limited). These cytokines, in turn, promote further IC acidification through their effects on mt function, creating a Tier 2 amplification loop between pH and inflammation that operates across multiple tissue types, including skeletal muscle, adipose tissue, liver, and pancreatic islets. The iNOS/eNOS-derived nitric oxide axis represents an additional pH-sensitive inflammatory effector [198,227], whose contribution to endothelial dysfunction and tissue inflammation in T2D is mechanistically plausible (**Supported** in vascular models) but whose relative magnitude compared to cytokine-driven inflammation in human CMAD is **Speculative**.

The third signaling axis is the Bcl-2/Bax mt apoptotic pathway. The balance between pro-apoptotic (Bax, BNIP3) and anti-apoptotic (Bcl-2, Bcl-xL) family members is directly modulated by pHi [63,64,65]. Under acidic conditions, Bax channel-forming activity increases markedly, demonstrated to be eightfold greater at pH 4.0 than at pH 7.5 in vitro [64] (**Supported** in vitro; the pH 4.0 condition substantially exceeds CMAD severity, and the magnitude of this effect at pH 6.9–7.1 relevant to CMAD has not been characterized). Bax pore formation in the mt outer membrane releases cytochrome c, which combines with Apaf-1 and procaspase-9 to form the apoptosome; caspase-9 activation is optimal at pH 6.3–6.8 in vitro [59] (**Supported** in vitro; these pH values are at the lower margin of or below typical CMAD pHi values, and direct activation of this mechanism specifically by CMAD-range acidosis in human diabetic tissue is **Speculative**). The downstream effector caspase-3 is activated via procaspase-3 maturation facilitated by acidification [60,61] (**Supported** in vitro). Across tissues including pancreatic β-cells, hepatocytes, skeletal myocytes, and neurons, convergent activation of this pathway under conditions of chronic mild acidosis, potentiated by co-existing oxidative stress, ER stress, and glucotoxicity in T2D, contributes to the progressive, multi-tissue apoptosis that characterizes advanced disease, though the independent contribution of pH to this process relative to co-operating pro-apoptotic stimuli cannot currently be quantified.

**Tier 3—Organ-Level Metabolic Consequences: Convergence and Amplification**

The third tier represents the tissue-specific integration of Tier 1 and Tier 2 disruptions into the organ-level pathology of T2D. At this tier, pH-mediated signaling dysfunction does not operate in isolation but converges with, and reciprocally amplifies, hyperglycaemia, dyslipidaemia, oxidative stress, and chronic inflammation, the established drivers of T2D pathology. The framework does not assert that CMAD is the primary driver of these outcomes; rather, it proposes that CMAD is a mechanistically coherent, biologically active contributor whose removal from the conceptual model of T2D leaves important aspects of the disease inadequately explained.

In skeletal muscle, the convergence of HKII suppression, PFK-1 pH-inhibition, PDC attenuation, histidyl dipeptide buffer depletion, and Akt-mediated GLUT4 impairment produces a tissue that is simultaneously glucose-intolerant, metabolically acidotic, proteolytically active, and progressively apoptotic, a phenotype consistent with the IR, lactate accumulation, protein catabolism, and sarcopenia documented in human T2D [82,83,112,113,119] (**Established** that these features coexist in human T2D; their attribution specifically to pH-mediated Tier 1–2 mechanisms is **Supported** at the pathway level but **Speculative** in terms of relative quantitative contribution). In the liver, acidosis-driven impairment of NHE-mediated pHi defense, combined with IR-associated lipid accumulation and inflammatory cytokine release, promotes the hepatocellular phenotype of nonalcoholic steatohepatitis, including stellate cell activation, fibrogenesis, and hepatocyte apoptosis [102,103,104,106] (**Supported**: pH–lipid–inflammation convergence demonstrated in hepatocyte models; CMAD-specific human liver pH data absent). In pancreatic β-cells, the paradoxical alkalinization of pHi against a background of interstitial acidosis creates a unique pH microenvironment that initially enhances and subsequently destabilizes insulin secretory function, ultimately converging on mt dysfunction, ER stress, and apoptotic β-cell loss [53,91,92,93,94] (**Supported** in rodent and in vitro models; direct human in vivo evidence absents). In adipose tissue, lipolysis-driven IC acidification, impaired insulin–NHE alkalinization, and cytokine-mediated metaflammation constitute a pH-linked inflammatory phenotype consistent with the adipose tissue dysfunction of T2D [125,126,127] (**Speculative** in the absence of direct human adipocyte pH measurement in T2D).

**Bidirectional Amplification and the Self-Perpetuating Nature of CMAD in T2D:** A defining feature of the proposed framework is that the three tiers do not operate as a unidirectional cascade but are connected by multiple feedback loops that are self-amplifying once established. Tier 1 glycolytic enzyme suppression increases lactate production, which deepens IC acidosis and further suppresses Tier 1 enzyme activity. Tier 2 inflammatory signaling drives mt dysfunction, which generates additional protons and deepens acidosis, re-engaging Tier 1 effectors. Tier 3 organ-level metabolic consequences, IR, sarcopenia, hepatic steatosis, β-cell failure, each independently promote anaerobic metabolism, mt dysfunction, and inflammation, all of which feedback to increase the systemic and tissue acid burden. This architecture of mutually reinforcing feedback explains why T2D is progressive, why correction of any single node produces incomplete clinical benefit, and why a multi-target strategy addressing CMAD alongside hyperglycaemia and inflammation may offer superior disease modification, though this last inference, while mechanistically coherent, remains **Speculative** in the absence of adequately designed interventional trials, as detailed in Section 4 and Section 5.2.

The bidirectional relationship between IR and CMAD deserves particular conceptual clarification. Whether acidosis precedes and drives IR, or whether IR precedes and drives acidosis, cannot currently be resolved with available human longitudinal data, and the manuscript does not claim otherwise. What the evidence does support is a mechanistically coherent cycle operating in both directions simultaneously. Acidosis disrupts the PI3K/Akt signaling pathway downstream of the insulin receptor, directly impairing GLUT4 translocation and glucose uptake [6]. In addition, CMA suppresses the activity of rate-limiting glycolytic enzymes, including phosphofructokinase-1 and the pyruvate dehydrogenase complex [81,111], diverting pyruvate toward anaerobic lactate production and deepening the acid burden independently of any antecedent IR. Conversely, IR diverts glucose flux toward anaerobic pathways [7,8], drives mt dysfunction and its associated proton generation [50], and impairs the insulin-stimulated NHE-mediated alkalinization that normally helps defend pHi [127]. Critically, experimental correction of acidosis with NaHCO_3_ and alkaline supplementation has been shown to improve insulin sensitivity in both clinical and preclinical settings [54], providing interventional evidence that acidosis can function upstream of IR, at least in certain contexts. The appropriate conceptual model is therefore not a linear causal chain but a self-amplifying loop, analogous to the well-established and widely accepted bidirectional relationship between inflammation and IR in T2D. The latter relationship, the field has productively investigated for decades without demanding that one process be definitively upstream of the other. Identifying the conditions under which acidosis or IR initiates this cycle in specific patient subgroups represents an important and tractable question for future longitudinal and interventional research.

A legitimate and important question concerns whether the magnitude of pH alterations documented in T2D is sufficient to generate the systemic biological consequences described in this review. Three lines of reasoning support the biological plausibility of significant effects at the pH ranges observed in CMAD. First, living cells operate within an extraordinarily narrow pHi window of less than 0.1 units [228], and the biochemical machinery of the cell is calibrated to this precision. A deviation that appears numerically modest on an absolute scale can therefore represent a substantial departure from the functional optimum. This is borne out experimentally: a fall of as little as 0.10 pH units in hepatocyte pHi suppresses glucose synthesis by 70% and urea synthesis by 50% [16], phosphofructokinase-1 activity changes markedly across the normal physiological pH range [81], and both DNA polymerase activity and protein synthesis rates exhibit steep pH dependence within the same narrow window [77,79]. These findings demonstrate that the cellular response to pH is not linear or proportional; it is steep and threshold-sensitive, operating precisely in the range of deviations observed in T2D. Second, the distinguishing feature of CMAD is not severity but chronicity. Unlike the acute, severe acidosis encountered in critical illness, the acid–base imbalance in T2D is mild but unrelenting, sustained across years to decades of disease. Chronic low-grade acidosis elicits maladaptive responses, including sustained caspase sensitization, progressive depletion of histidyl dipeptide buffers, cumulative impairment of insulin signaling cascades, and potentially epigenetic remodeling of pH-responsive gene networks [82]. These effects are not replicated in acute experimental models, and their cumulative toll cannot be inferred from short-term pH perturbation studies. Third, systemic measurements of acid–base status in T2D, including serum bicarbonate and venous blood pH, likely represent a significant underestimate of the acid burden at the tissue level. The fourfold increase in intramuscular lactate in diabetic skeletal muscle [112], the measurable decline in muscle pHi [82], the acidification of bone marrow sufficient to activate osteoclastogenesis [41,181], and the well-documented reductions in salivary and urinary pH all indicate that compartment-specific acidosis can be substantially more pronounced than systemic markers suggest. Taken together, these considerations support the biological plausibility of CMAD-driven pathology at the pH ranges observed clinically, though we acknowledge that formal dose–response characterization between the degree of CMAD and specific organ outcomes in human cohorts remains an important unmet research need.

Importantly, this review also exposes significant gaps in our current understanding. Direct measurements of tissue-specific pHi and pHn in diabetic patients remain sparse, particularly for adipose tissue, nervous system components, and various epithelial surfaces. The temporal dynamics of pH changes during diabetes progression, the heterogeneity of acidosis across different patient populations, and the quantitative contribution of CMAD relative to other pathophysiological mechanisms require systematic investigation. Furthermore, the optimal strategies for pH monitoring in clinical practice and the development of targeted therapies that address tissue-specific pH dysregulation represent important frontiers for translational research.

Looking forward, we propose a paradigm shift in conceptualizing T2D pathophysiology, one that positions CMAD not as a mere epiphenomenon of metabolic derangement but as a feature that both drives and is driven by the disease process. This reconceptualization suggests several research priorities: (1) comprehensive mapping of pH dynamics across tissues and subcellular compartments in diabetic versus non-diabetic states; (2) elucidation of the molecular mechanisms linking pH disturbances to specific pathological outcomes; (3) development of biomarkers for CMAD severity and tissue-specific acidosis; (4) design of clinical trials examining pH-targeted interventions as adjunctive or even primary therapeutic strategies; and (5) investigation of whether early pH correction might prevent or delay T2D onset in high-risk individuals.

### 5.2. Current Evidence and Limitations

The preceding sections of this review have presented a mechanistic framework supported by convergent but largely indirect evidence. The specific limitations constraining the strength of each inferential step are detailed below, and readers are encouraged to weigh these limitations carefully when evaluating the framework as a whole. Several important limitations constrain the strength of the inferences drawn. First, direct measurements of tissue-specific pHi and pHn in human diabetic cohorts remain scarce. For main metabolic tissues, including adipose tissue and components of the nervous system, such data are essentially absent, and the pH changes attributed to these tissues in the context of T2D are extrapolated from non-diabetic physiological studies, animal models, or indirect biomarkers. Second, much of the mechanistic evidence linking acidosis to cellular dysfunction, including effects on enzyme kinetics, apoptotic pathways, ion conductance, and macromolecule synthesis, derives from in vitro experiments conducted under acute acidification conditions that may not faithfully replicate the mild, chronic acidosis characteristic of CMAD. Caution is therefore warranted when extrapolating these findings to the in vivo human setting. Third, the associations described between CMAD and organ-level pathology in T2D are predominantly correlational. While the alignment between the biological effects of acidosis and the clinical features of T2D is striking, correlation does not establish causation, and the relative contribution of CMAD to each pathological process, compared to hyperglycemia, lipotoxicity, oxidative stress, and other established mechanisms, remains unquantified. Fourth, the bidirectional relationship between CMA and T2D, though conceptually compelling, has not been systematically disentangled. It is currently unclear to what extent CMA precedes and drives T2D pathology versus arising as a consequence of it, and whether the two processes are separable in clinical practice. Finally, evidence supporting acid-neutralization as a therapeutic strategy, while encouraging, remains preliminary and is drawn from heterogeneous studies of variable quality, many of which were not designed to test the CMAD hypothesis specifically.

A critical evidentiary limitation that requires explicit acknowledgment concerns the quality and source of pH data for three tissues central to the proposed CMAD framework: adipose tissue, the central nervous system, and pancreatic β-cells. For none of these tissues does a body of direct, in vivo, human pH measurement data currently exist in the diabetic context, and the mechanistic assertions made in this review regarding these tissues rest on evidence of varying and generally indirect quality. For adipose tissue, no published measurements of human adipocyte pHi or interstitial pHn in the diabetic state have been identified in the literature, as explicitly acknowledged in Section 3 (part iv). The inference that adipocyte pHi declines in T2D is derived by extrapolation from the known alkalinizing effect of insulin on adipocytes via NHE activation [127], the parallel acidification documented in liver and skeletal muscle under IR conditions, and the general association between mt dysfunction and intracellular proton accumulation, none of which constitutes direct measurement. For the central nervous system, direct neuronal pHi measurement in living human subjects is not currently achievable by any clinically or ethically feasible method. The available evidence derives from cerebrospinal fluid pH studies, animal models of diabetic encephalopathy, and indirect neuroimaging biomarkers such as lactate-to-pyruvate ratios; this evidence is suggestive but cannot be considered confirmatory of the CNS acidification posited in Section 3 (part vi). For pancreatic β-cells, the proposed increase in β-cell pHi in T2D is supported by in vitro data and rodent model studies, and is mechanistically elaborated in the recent work of Fang et al. [95], but has not been directly confirmed by in vivo human measurement. All assertions regarding pH dynamics in these three tissues should therefore be understood as mechanistically informed hypotheses rather than facts. This evidentiary gap itself constitutes one of the most compelling arguments for the research agenda proposed in this review: the development of non-invasive or minimally invasive tools for tissue-specific pH monitoring in human cohorts, including magnetic resonance spectroscopy-based pH mapping, pH-sensitive positron emission tomography tracers, and implantable nanosensor technologies, represents a priority need if the CMAD hypothesis is to be rigorously tested in the tissues where it matters most.

The convergence of evidence presented in this review supports the conclusion that CMA warrants serious consideration as a contributor to T2D pathophysiology and that pH homeostasis is a potentially important therapeutic target that merits rigorous formal investigation [3,54]. However, this conclusion must be stated with appropriate circumspection. The preliminary observations from acid-neutralization studies provide biologically plausible proof of concept rather than clinical validation [203,204,206], and they do not constitute a sufficient evidentiary basis for changes to clinical practice. The path from the proposed mechanistic framework to an actionable therapeutic strategy requires prospective trials with pre-specified pH-related endpoints, careful patient selection accounting for comorbidities and polypharmacy [215,221,222]. Also requires systematic safety monitoring for overcorrection and organ-specific adverse effects [211,214] and direct tissue-level pH measurement to confirm that systemic interventions achieve the intended compartment-specific targets [225]. Until such trials are conducted, pH homeostasis should be regarded as a compelling, testable hypothesis rather than an established therapeutic principle, and clinicians should not modify T2D management based on the currently evidenced CMAD framework.

These limitations do not diminish the value of the proposed framework, which offers a unifying perspective with genuine explanatory power and testable predictions. Rather, they underscore the need for prospective studies with direct pH monitoring, mechanistic human cohort data, and rigorously designed clinical trials before CMAD can be elevated from a compelling hypothesis to an established pillar of T2D pathophysiology.

## 6. Conclusions

Chronic metabolic acidosis of diabetes represents an underappreciated but mechanistically coherent contributor to T2D pathophysiology. The three-tier framework presented here illustrates how mild, sustained reductions in intracellular pH dysregulate glycolytic enzymes, ion transporters, and buffering proteins at the molecular level propagate disruption through insulin signaling, inflammatory and apoptotic cascades, and converge with established co-operating mechanisms to produce organ-level metabolic dysfunction. Critically, this relationship is bidirectional: insulin resistance deepens acidosis, which in turn amplifies insulin resistance, creating a self-perpetuating cycle with clear parallels to the well-characterized inflammation-IR axis.

The evidence reviewed ranges from established human data to preclinical inference, and this heterogeneity demands interpretive restraint. No pH-directed intervention has been tested in an adequately powered trial specifically targeting T2D outcomes, and systemic alkalinization carries clinically meaningful risks that must inform any future interventional design. Nevertheless, pH homeostasis in T2D constitutes a testable hypothesis with strong molecular rationale. Advances in tissue pH mapping, pH-sensitive imaging, and nanosensor technology now make prospective validation feasible. If confirmed, targeted pH correction could offer a mechanistically novel adjunct to standard T2D management, acting simultaneously across multiple tiers of pathophysiology rather than at isolated downstream effectors.

## Figures and Tables

**Table 1:** The pH of normal body fluids, excretions, and pH changes in T2D.

Fluid	Normal			T2D		
	pH	Source	References	pH Change	Source	References
ICF	7.0–7.4	Eukaryotes	[11]	↔	Human	[12]
ISF	6.6–7.6	Human	[12]	↓	Human	[12]
CSF	7.30–7.36	Human	[13]	↔	Human	[14]
Pleural fluid	Approx. 7.6	Human	ECLI 2022	NK		
Peritoneal fluid	7.5–8.0	Human	[15]	NK		
Synovial fluid	7.31–7.64 7.4–7.8	Human Human	[16,17]	NK		
Tear	6.5–7.6 7.50 (±0.23)	Human Human	[18,19]	NK		
Vitreous humor	7.0–7.4 7.25 ± 0.02	Human	[20]	↔	Human	[20]
Aqueous humour	7.1–7.4	Human	[21]	NK		
Saliva	6.2–7.6	Human	[22]	↓	Human	[23]
Sweat	4.0–6.8	Human	[16]	NK		
GB bile CBD bile	6.80–7.65 7.50–8.05	Human Human	[24]	NK NK		
Gastric secretion	1.0–3.5	Human	[25]	↑	Human	[26]
Intestine content	1.7–9.0	Human Human	[25,27]	↓		
Vaginal secretions	3.5–4.0	Human	[16]	↓	Human	[28]
Seminal fluid	7.2–8.0	Human	[16]	↓	Human	[29]
Urine (UB)	4.8–8.4	Human	[30]	↓	Human	[30]
Stool	6.5–7.5	Human	CTC, 2022	↓		NK

NK: not known; arrow direction indicates the pH changes in T2D with reference to normal values: increase (↑), decrease (↓), or no change (↔); ICF: intracellular fluid, ISF: interstitial fluid, CSF: cerebrospinal fluid, GB: gall bladder, CBD: common bile duct, UB: urinary bladder, ECLI: Exeter Clinical Laboratory International, CTC: https://www.pathology.med.umich.edu/handbook/#/details/718 (accessed on 20 January 2026).

**Table 2:** Normal tissue-specific intracellular fluids pH, and changes in T2D.

Tissue (ICF)	Normal pH			T2D		
	7.0–7.4	Source	References	pH Change	Sources	References
Brain	7.2	Human	[16]	NK		
Neuron cells	~7.03–7.46	Rat	[35]	NK		
Eye retinal neuron	NK			↓	Non-human	[3]
Heart	7.00 ± 0.06 7.1–7.2	Human Human	[16,36]	NK		
Lung	6.7	Human	[16]	NK		
Liver	7.0 7.0 (6.8–7.2)	Human In vitro	[16,37]	NK		
Pancreas β-cells	7.5–8 8.0–8.3	Human In vitro *	[16,38]	NK ↑	In vitro *	[38]
Kidney	7.0–7.3 5–7.3	Human	[16]	NK		
Adipocytes			NK	NK		
Skeletal muscle	7.00 ± 0.06	Human	[39]	↓	In vivo/vitro *	[40]
Bones	7.4	Human	[16]	↓	Mice	[41]
Skin surface	4.7 5.4–5.9	Human Human	[42,43]	↑	Human	[44]

Note: Some pH values were obtained from experimental animals or in vitro from both (* Human & animal) (see references). NK: Not known. The direction of the arrows indicates an increase (↑) or decrease (↓).

## Data Availability

Data sharing not applicable—no new data generated.

## Supplementary Materials

The following supporting information can be downloaded at: https://www.mdpi.com/article/10.3390/biomedicines14040901/s1, Table S1: Acid-neutralizing drug therapies and supplements that are used in the management of chronic acidosis (systemic and local), and that are used or can be used in T2D. Table S2: Summary of systemic and tissue-specific pH values in type 2 diabetes (T2D) and comparison with experimental pH ranges used in cited studies.
