# Supplementary material for: Toll of Chronic Metabolic Acidosis at Molecular, Cellular, and Systemic Levels: A Conceptual Framework to Revisit Type 2 Diabetes (T2D) Pathophysiology
Source: Biomedicines. 2026 Apr 15;14(4):901. doi: 10.3390/biomedicines14040901 (PMC13113581; doi:10.3390/biomedicines14040901)
Supplement: Supplementary file 1 [file biomedicines-14-00901-s001.zip › biomedicines-4135127-supplementary.pdf]

Supplementary material

Table S1: Acid-neutralizing drug therapies and supplements that are used in the management of chronic acidosis (systemic and local), that are used or can be used in T2D.

Drug and Supplements Used for (disorder)	Action	Mechanism of action	Systemic/local (S/L) effect
Alkaline minerals: (T2D)	Alkaline addition	AN	S & L (Renal; stones, infections)
CAIs (MetSyn – trials)	Buffering	H_2CO_3	Eye: PRD
CALC nanosheets (T2D complications - trials)	AN	Immunotherapeutic	Bone: Osteoporosis
Chewing gum – sugar-free<br>Arginine (Arg) dentifrices (T2D dental complication)	Alkaline addition<br>Buffering	AN<br>Produce NH3 via ADS	Oral: infections, dental caries
CPT1 (T2D & MetSyn - trials)	Acid reduction (lactate)	FA oxidation Inhibition	Heart: Cardiac ischemia
HDAC4 inhibitors (T2D – trials)	Ion transporters (NHE6 expression)	Normalize ApoE4 by decreasing ES pH	Brain: cognitive disorders
NaHCO3+ Ca-gluconate (Bone disorder)	Alkaline addition	AN	Joints: Osteoarthritis
NOS-I (IR- trial)	Acid reduction	Inflammation inhibition	Immunity: Vasculitis
Sodium butyrate (NaB) (T2D – trial)	Multiple	Enhances GLP-1R, inhibit HDAC2, others	L (Liver: NAFLD/ NASH)L (muscles: sarcopenia)
PPIs (Gut disorders, T2D!!)	Acid reduction	blocking H^+/K^+ ATPase	S & L (GIT symptoms)
Veverimer (CKD – T2D!!)	Acid reduction	HCL binder block acid absorption	Kidney: CKD

AN: Acid neutralization; ADS: arginine deiminase system; CAIs: Carbonic anhydrase inhibitors; CALC: calcein functionalized calcium–aluminum-layered double hydroxide; CPT1: carnitine palmitoyltransferase inhibitors; ES: endosomal; HDAC4: H_2CO_3: carbonic acid; Histone deacetylase 4; ; PDR: proliferative diabetic retinopathy; NH3: ammonia; MetSyn: metabolic syndrome; IR: insulin resistance; CKD: chronic kidney disease; NASH: Non-alcoholic steatohepatitis; NAFLD: non-alcoholic fatty liver disease; HCL: hydrochloric acid; ; NOS-I: Nitric oxide synthase inhibitor PPIs: Proton pump inhibitors; T2D!!: Not known; T2D -trial: under investigation. T2D!!: logical assumption, needs to be proved or disproved.

Note: Details and sources (references) of all drug/therapy items are mentioned in the original review article (Giha, 2025).

Table S2: Summary of systemic and tissue-specific pH values in type 2 diabetes (T2D) and comparison with experimental pH ranges used in cited studies

Tissue / Compartment	Normal pH (reference range)	Reported / Inferred pH in T2D	Direction of Change	pH Range Used in Cited Experimental Studies	Experimental Context (what was measured/manipulated)	Key References
**SYSTEMIC / BLOOD**						
**Arterial blood**	7.35–7.45	Low-normal to mildly reduced; chronic low HCO_3^- (serum HCO_3^- often 18–22 mEq/L in T2D vs. 22–26 mEq/L in controls)	↓ (mild)	pH 7.10–7.35 (moderate acidosis models)	Enzyme kinetics, PI3K/Akt signalling disruption studies	[4, 5, 6]
**Venous blood**	7.32–7.43	Parallels arterial reduction; lactate elevated up to fourfold in skeletal muscle effluent	↓ (mild)	pH 7.10–7.30	In vitro IR signalling, GLUT4 translocation assays	[6, 87]
**INTRACELLULAR pH (pHi) – KEY METABOLIC TISSUES**						
**Hepatocytes (liver)**	7.00 ± 0.03	Presumed decreased; even 0.10 pH unit fall inhibits glucose synthesis 70%, urea synthesis 50%. IR and T2D logically reduce pHi via impaired insulin-NHE axis	↓ inferred	pH 6.90–7.00 (acute acidosis models)	Hepatocyte glucose/urea synthesis, lipid metabolism, apoptosis studies	[69, 70, 71, 80]
**Skeletal muscle (myocytes)**	7.00 ± 0.06	Measurably decreased in T2D; fourfold increase in intramuscular lactate; histidyl dipeptide buffer depletion contributes	↓ confirmed (indirect)	pH 6.80–7.00	PFK-1 activity assays; IRS-PI3K-AKT signalling; actin/myosin contractility; carnosine buffer studies	[25, 54, 85, 87, 88, 91, 94, 95, 96]
**Pancreatic β-cells**	~7.20–7.35 (estimated; direct human data absent)	INCREASED pHi in T2D (paradoxical alkalinisation); low interstitial pHn coexists. Based on in vitro and rodent data; no direct human in vivo measurement confirmed	↑ (pHi); ↓ (pHn)	pH 7.35–7.55 (alkaline range); pH 6.80–7.10 (interstitial acidosis models)	K^+-channel conductance, Ca^{2+} exocytosis, apoptosis (caspase-9 optimum pH 6.3–6.8), insulin secretion studies	[27, 32, 61, 62, 68]
**Adipocytes (adipose tissue)**	Not reported in humans	No direct human measurement available; pHi decline inferred from IR-impaired NHE alkalinisation and parallel changes in liver/muscle	↓ inferred (extrapolated)	pH 6.80–7.10	NHE-mediated FFA esterification; lipolysis-induced acidification; insulin alkalinisation of adipocytes	[100, 102]
**INTRACELLULAR pH (pHi) – NERVOUS SYSTEM**						
**Neurons / CNS (general)**	~7.05–7.30 (cell-type dependent; hippocampal neurons ~7.05)	No direct in vivo human measurement in T2D; CNS acidification inferred from lactate:pyruvate neuroimaging ratios, CSF studies, and animal models of diabetic encephalopathy	↓ inferred	pH 6.80–7.20	Neuronal excitability, action potential threshold, K^+/Ca^{2+} channel pH-sensitivity, neurotransmitter receptor function	[24, 127, 128, 129, 130, 131, 132]
**Cerebrospinal fluid (CSF)**	7.33 ± 0.02	Not directly reported in T2D; controlled by choroid plexus and glia; assumed to reflect mild systemic acidosis with buffering lag	↓ probable	pH 7.20–7.30	CNS pH control; neurodegeneration models (Alzheimer's, Parkinson's, vascular dementia)	[131, 135, 136, 137, 138]

Tissue / Compartment	Normal pH (reference range)	Reported / Inferred pH in T2D	Direction of Change	pH Range Used in Cited Experimental Studies	Experimental Context (what was measured/manipulated)	Key References
**INTERSTITIAL / EXTRACELLULAR pH (pHn) – SPECIFIC ORGANS**						
**Skeletal muscle interstitium**	~7.40	Reduced in proportion to lactate accumulation and proton efflux; quantitative T2D-specific data limited	↓ (indirect evidence)	pH 7.00–7.35	MCT-mediated H^+ efflux; NHE activity in muscle; acidosis–IR crosstalk	[17, 81, 87, 94]
**Pancreatic interstitium (pHn)**	Not reported	Presumed acidic due to increased metabolic stress, mitochondrial dysfunction, and proton efflux from β-cells; no published measurement	↓ presumed	pH 6.80–7.10	β-cell dysfunction, ion channel disruption, apoptosis cascade activation	[27, 65]
**Synovial fluid (joints)**	~7.4	Acidic in inflammatory arthropathy; correlates with radiological joint destruction; T2D-associated arthropathy likely shares this profile	↓ (arthropathy context)	pH 6.80–7.20	Chondrocyte GAG synthesis (optimum pH 7.2); cartilage proteoglycan integrity	[175, 178, 179, 180]
**Bone marrow**	~7.35–7.40	Acidic in T2D; sufficient to activate osteoclasts via TRPV1/TRPV4; nanotechnology-based BM pH correction improves bone outcomes in preclinical models	↓ confirmed (preclinical)	pH 6.80–7.20	Osteoclast activation (RANKL/OPG/RANK axis); TRPV1/TRPV4 stimulation; osteoblast inhibition	[161, 162, 163, 164, 165, 169]
**SECRETED / EXCRETED FLUIDS**						
**Urine**	4.6–8.0 (mean ~6.0)	Markedly acidic in T2D (often pH <5.5); independently associated with uric acid nephrolithiasis; persists after correction for diet, BMI, renal function	↓ confirmed (clinical)	pH <5.5 (stone-forming threshold)	Uric acid solubility and crystallisation; renal acid excretion; ammonium/urea handling	[147, 148, 149, 150, 151]
**Saliva**	6.2–7.6 (mean 6.7)	Significantly lower in T2D (reported 6.5 vs. 7.88 in controls); attributed to reduced salivary flow, low HCO_3^-, and microbial acidogenesis	↓ confirmed (clinical)	pH 5.5–6.5	Aciduric bacterial growth; dental caries development; oral mucosal defence	[170, 171, 172, 173]
**Gastric fluid**	1.0–3.5	Increased (less acidic) in T2D, especially in gastroparesis; fasting gastric pH elevated vs. healthy controls	↑ (reduced acidity)	pH 1.0–3.5 (normal) vs. >4.0 (T2D/gastroparesis)	Drug solubilisation and absorption modelling; H^+-ATPase activity in gastric parietal cells; GM composition	[105, 106, 107, 119, 120]
**Skin surface (stratum corneum)**	4.0–5.0 (mean ~4.7)	More alkaline in T2D (>5.0); disrupts antimicrobial barrier; predisposes to Candida intertrigo in intertriginous areas	↑ (more alkaline)	pH 4.0–4.5 (normal barrier) vs. pH 6.5–9.0 (candidal hypha growth optimum)	Skin barrier function assays; Candida hypha germination; bacterial flora adhesion	[152, 153, 154, 155, 156, 157, 158, 159]
**Pancreatic secretions**	8.0–8.3	Alkalinity retained or modestly reduced; contributes to duodenal neutralisation; T2D-specific data limited	Uncertain	pH 7.8–8.3	Bicarbonate secretion modelling; duodenopylorogastric reflex studies	[3, 10]
**ORGANELLE-LEVEL pH (illustrative; not T2D-specific unless stated)**						
**Mitochondrial matrix**	~8.0	Depolarisation and pH gradient dissipation documented in T2D mitochondria; exact ΔpH not reported in human T2D	↓ gradient (inferred)	pH 7.0–8.0	Oxidative phosphorylation efficiency; Cyt c release; apoptosome assembly; Bcl-2/Bax pore formation	[13, 15, 22, 37, 38, 48]

Tissue / Compartment	Normal pH (reference range)	Reported / Inferred pH in T2D	Direction of Change	pH Range Used in Cited Experimental Studies	Experimental Context (what was measured/manipulated)	Key References
**Lysosomes**	~4.5	Lysosomal dysfunction reported in T2D; exact pH not measured; autophagy dysregulation implies altered lysosomal acidification	↑ probable (less acidic, dysfunction)	pH 4.5–5.0	DNase II activation (pH <7.0 required); autophagy; LEI translocation	[13, 14, 25, 30, 31]
**Golgi apparatus**	6.0–6.7 (cis→trans gradient)	Not measured in T2D; expected perturbation secondary to ER stress and vesicular trafficking dysfunction	Uncertain	pH 6.0–7.0	Glycosylation processing; vesicle sorting; insulin granule maturation	[16]

Notes:

↓ = decreased relative to normal reference range; ↑ = increased (less acidic / more alkaline); pHi = intracellular pH; pHn = interstitial (neighbourhood) pH; ECF = extracellular fluid; ICF = intracellular fluid; IR = insulin resistance; T2D = type 2 diabetes mellitus; CMAD = chronic metabolic acidosis of diabetes; MCT = monocarboxylate transporter; NHE = Na^+/H^+ exchanger; PFK-1 = phosphofructokinase-1; PDC = pyruvate dehydrogenase complex; IRS = insulin receptor substrate; PI3K = phosphatidylinositol-3 kinase; GLUT4 = glucose transporter 4; GAG = glycosaminoglycan; GM = gut microbiota; BM = bone marrow; RANKL = receptor activator of nuclear factor kappa-B ligand; OPG = osteoprotegerin; TRPV = transient receptor potential vanilloid; Cyt c = cytochrome c; ER = endoplasmic reticulum.

† Direct in vivo human pH measurement in T2D confirmed: urine, saliva, gastric fluid, skin surface, arterial/venous blood. All other tissue pHi values in T2D are inferred from indirect biomarkers, animal models, ex vivo data, or extrapolation from non-diabetic physiological studies, as explicitly stated in each row and in subsection 5.1 of the manuscript.

‡ Experimental pH values represent the range employed in the in vitro, ex vivo, or animal studies cited in the corresponding reference column. These experimental conditions were not always designed to replicate CMAD specifically and may employ more extreme pH perturbations than those observed in T2D. Readers should interpret the mechanistic evidence accordingly.

§ All reference numbers correspond to the manuscript's reference list. References [186]–[209] are new references added in response to reviewer comments.
